# Metastatic renal cell carcinoma with occult primary: a multicenter prospective cohort

**DOI:** 10.1038/s41698-024-00648-0

**Published:** 2024-07-18

**Authors:** Nicolas Jacquin, Ronan Flippot, Julien Masliah-Planchon, Guillaume Grisay, Riwan Brillet, Célia Dupain, Maud Kamal, Isabelle Guillou, Nadège Gruel, Nicolas Servant, Pierre Gestraud, Jennifer Wong, Vincent Cockenpot, Andreia Goncalves, Janick Selves, Hélène Blons, Etienne Rouleau, Olivier Delattre, Claire Gervais, Christophe Le Tourneau, Ivan Bièche, Yves Allory, Laurence Albigès, Sarah Watson

**Affiliations:** 1 INSERM U830, Diversity and Plasticity of Childhood Tumors Lab, PSL Research University, Institut Curie Research Center, Paris, France; 2 Department of Medical Oncology, Institut Godinot, Reims, France; 3 Department of Cancer Medicine, Institut Gustave Roussy, Université Paris-Saclay, Villejuif, France; 4 Somatic Genetic Unit, Department of Genetics, Institut Curie Hospital, Paris, France; 5 Clinical Bioinformatic Unit, Department of Diagnostic and Theragnostic Medicine, Institut Curie Hospital, Paris, France; 6 Department of Drug Development and Innovation (D3i), Institut Curie, Paris, France; 7 Department of Translational Research, Institut Curie Hospital, Paris, France; 8 INSERM U900, CBIO-Centre for Computational Biology, Institut Curie Research Center, Mines ParisTech, Paris, France; 9 Department of Pathology, Institut Curie Hospital, Paris, France; 10 Department of Pathology, University Hospital of Toulouse (IUCT), Toulouse, France; 11 Department of Biochemistry, Pharmacogenetics and Molecular Oncology, Georges Pompidou European Hospital, APHP, Paris, France; 12 PRISM Center for personalized medicine, Gustave Roussy Cancer Center, Villejuif, France; 13 Department of Medical Oncology, Georges Pompidou European Hospital, APHP, Paris, France; 14 INSERM U900, Institut Curie, Saint-Cloud, France; 15 Paris-Saclay University, Paris, France; 16 Department of Genetics, Institut Curie Hospital, INSERM U1016, Université Paris Cité, Paris, France; 17 Department of Pathology, Institut Curie Hospital, Saint-Cloud, France; 18 Université Versailles St-Quentin, Université Paris-Saclay, Montigny-le-Bretonneux, France; 19 Department of Medical Oncology, Institut Curie Hospital, Paris, France

**Keywords:** Molecular medicine, Cancer of unknown primary, Cancer genomics

## Abstract

Metastatic carcinoma of presumed renal origin (rCUP) has recently emerged as a new entity within the heterogeneous entity of Cancers of Unknown Primary (CUP) but their biological features and optimal therapeutic management remain unknown. We report the molecular characteristics and clinical outcome of a series of 25 rCUP prospectively identified within the French National Multidisciplinary Tumor Board for CUP. This cohort strongly suggests that rCUP share similarities with common RCC subtypes and benefit from renal-tailored systemic treatment. This study highlights the importance of integrating clinical and molecular data for optimal diagnosis and management of CUP.

## Manuscript

Cancers of unknown primary (CUPs) are classically subdivided into two prognostic subsets according to their clinical and pathological presentation. Tumors of the favorable subset show similarities with metastatic cancers of known primary and benefit from tissue-specific treatments^1^. Among them, metastatic carcinoma of presumed renal origin (rCUP) has recently emerged as a new tumor entity^2^. However, only limited clinical case series have been reported and their molecular features as well as optimal therapeutic management remain unknown^3–6^.

We report a comprehensive clinical, pathological and molecular characterization of rCUP as well as outcomes on therapy in patients prospectively identified through the French national multidisciplinary tumor board for CUPs (NatCUPMTB).

### Clinical presentation

Out of 270 patients presented at NatCUPMTB since June 2020, 25 (9%) were identified as rCUP and included in this study (Supplementary Table 1). The diagnosis of rCUP was proposed by the MTB based on the combination of clinical and pathological features for all 25 patients, as well as on additional genomic and/or transcriptomic characteristics for 16/25 (64%) patients.

Median age at diagnosis was 58 years (range 29-85), and 18/25 (72%) were men. The most common metastatic sites at diagnosis were the bone (*n* = 16/25, 64%) and lymph nodes (*n* = 16/25, 64%). Five patients (20%) presented with oligometastatic disease at diagnosis, and 20/25 (80%) patients with diffuse metastases in at least two distinct sites.

### Molecular analyses

**Fig. 1:**
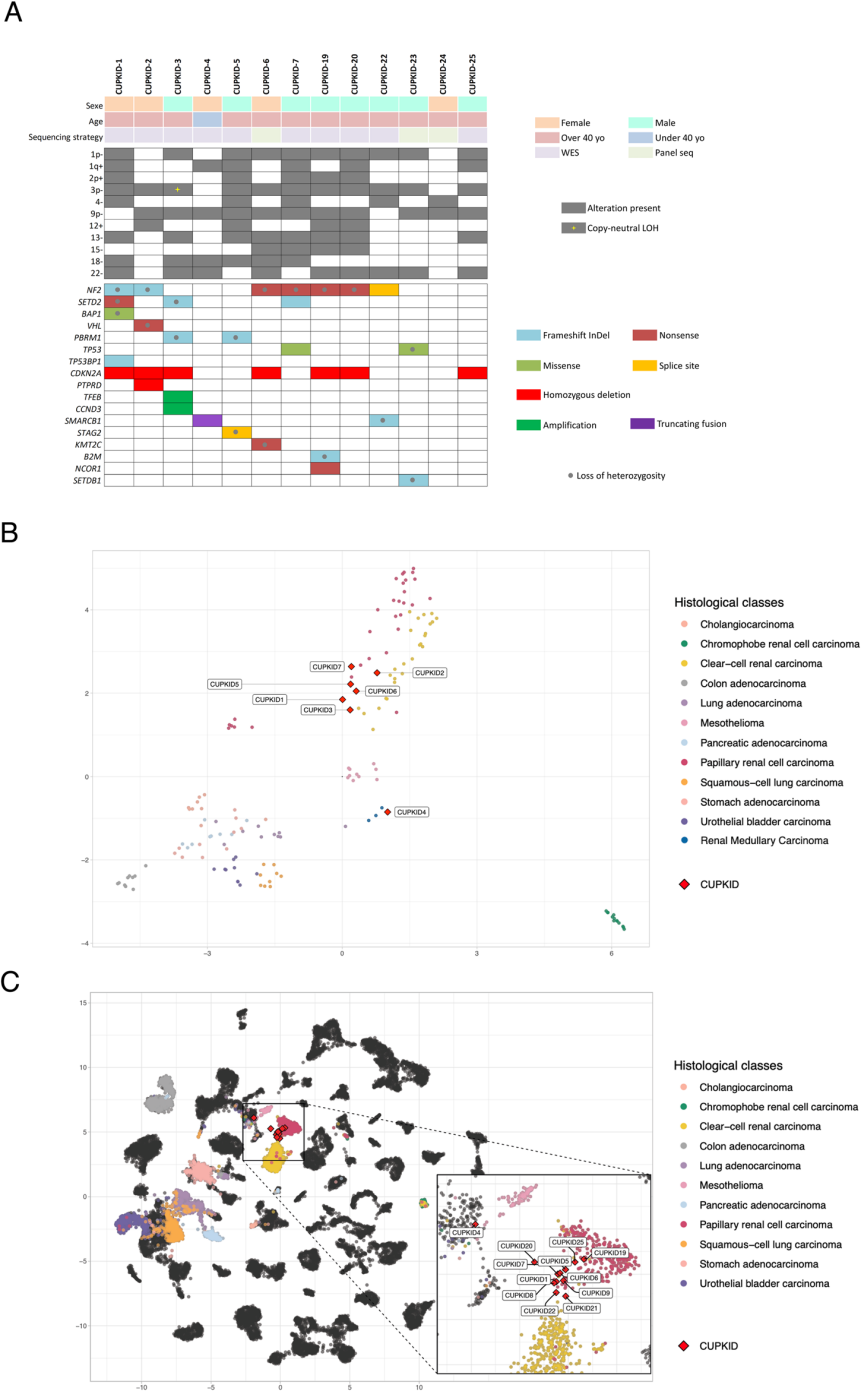
Molecular landscape of CUPKID samples. **A** Genomic alterations identified by WES/WGS or targeted DNA sequencing (panel Seq) in 13 patients. **B** DNA methylation profiles of 7 tumor samples compared to a cohort of metastatic carcinomas of renal, lung, gastrointestinal and biliary origin using unbiased clustering based on the 5000 most variable CpG sites. Each CUPKID sample is highlighted with a red diamond. **C** RNAseq transcriptomic profiles of 12 tumors plotted on the Uniform Manifold Approximation and Projection (UMAP) representation of the TransCUPtomics classifier. Each CUPKID sample is highlighted with a red diamond. Annotated tumor and normal tissue samples used to trained the classifier are represented as dots, with specific classes of interest highlighted in colors and other classes in dark grey.

Whole exome sequencing (WES) and Whole Genome Sequencing (WGS) were performed on tumor DNA from cryopreserved tissues and matched germline DNA from 10 rCUP patients for detection of single nucleotide variations (SNV) and copy number variations (CNV). For 3 patients for which no frozen tumor sample was available, a targeted next generation sequencing DNA panel of 571 genes involved in solid tumors (Agilent SureSelect CD Curie CGP) was performed on FFPE samples (Fig. 1A and Supplementary Table 2). We identified recurrent inactivating mutations in tumor suppressor genes classically inactivated in kidney clear cell carcinoma (KIRC) and papillary carcinoma (KIRP)^7,8^ including *NF2* (*n* = 7, 54%), *SETD2* (*n* = 3, 23%), *PBRM1* (*n* = 2, 15%), *BAP1* (*n* = 1, 8%) and *VHL* (*n* = 1, 8%). Recurrent CNV commonly identified in common RCC subtypes included *CDKN2A/CDKN2B* homozygous deletions (*n* = 7, 60%), losses of chromosomes 1p (*n* = 10), 3p (*n* = 11), 9p (*n* = 11), 13 (*n* = 8), 18 (*n* = 6) and 22 (*n* = 9), and gains of 1q (*n* = 6), 2p (*n* = 5), and 12 (*n* = 4). Amplification of *TFEB*, a *SMARCB1* truncating fusion, and a *PRCC-TFEA3* fusion were detected in CUPKID-3, CUPKID-4, and CUPKID-24, respectively. The median tumor mutational burden was 1.7 mut/Mb (range 0.5-11.3), with absence of detectable mutational signature or microsatellite instability.

DNA methylation analyses were performed on tumor DNA from 7 patients using the Infinium Methylation EPIC microarrays v1.0 850 K (Illumina) and compared to metastatic carcinoma samples of renal, lung, gastrointestinal and biliary origins from the TCGA database and from public series (Supplementary Table 3)^9^. Unbiased clustering based on the 5000 most variable CpG sites showed that all 7 samples clustered together with specific RCC subtypes, including CUPKID-4 with RMC, supporting their common tissue of origin (Fig. 1B).

Finally, whole transcriptome sequencing (RNA-seq) was performed on tumor RNA from 12 rCUP samples for fusion detection and gene expression profiling. Gene expression data were analyzed using the TransCUPtomics classifier^10^, whose reference dataset includes public samples of KIRC, KIRP, and chromophobe renal cell carcinoma (KICH). Eleven out of 12 tumors were classified as KIRC or KIRP by TransCUPtomics according to their gene expression profiles (Fig. 1C and Supplementary Table 4). CUPKID-4, harboring a *SMARCB1*-truncating fusion, remained unclassified according to TransCUPtomics, in line with the absence of renal medullary carcinoma (RMC) in the reference dataset^9^.

### Pathological review

A centralized pathological review (*N* = 18) or local pathological review (*N* = 7) was carried out and integrated together with molecular features for definitive tumor classification (Supplementary Fig. 1 and Supplementary Table 5). This led to the modification of the initial suspected diagnosis in 15/25 (60%) samples. Seven samples were classified as KIRC, three samples as KIRP, one sample as RMC, one sample as *TFEB*-amplified RCC, and one sample as *TFE3*-rearranged RCC. Twelve samples showed morphological and molecular features concordant with their renal origin but lacked features specific of precise RCC subtypes and were termed as unclassified RCC (URCC).

### Treatment and follow-up

**Fig. 2:**
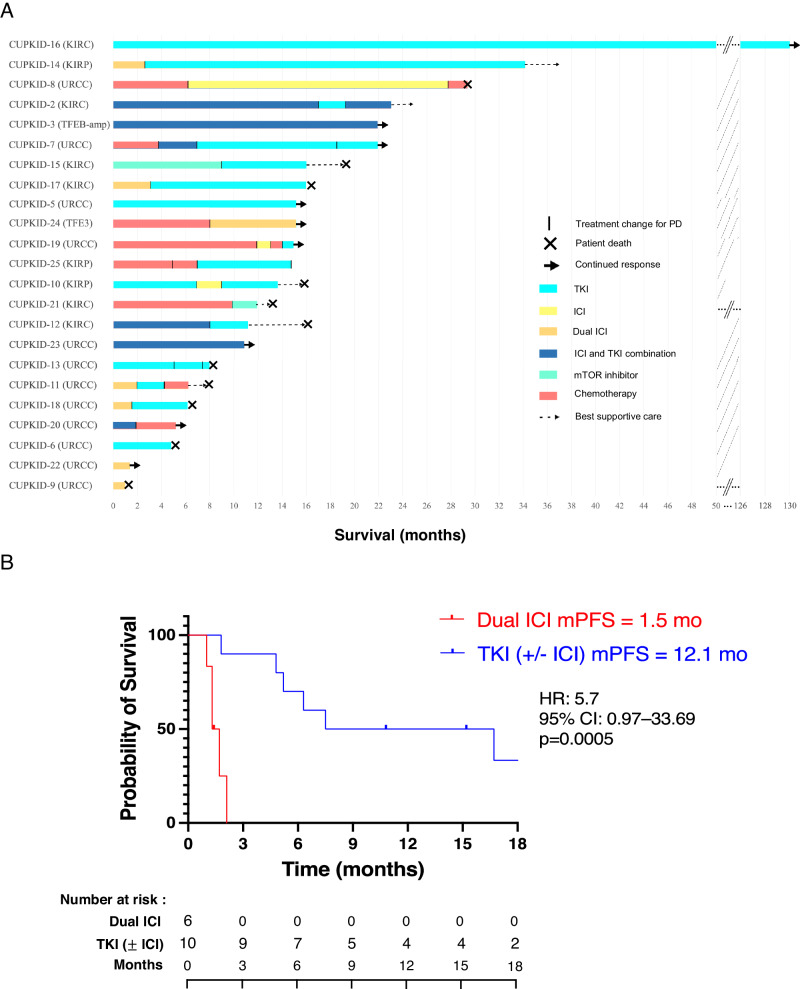
Therapeutic management and outcome of 23 patients treated with systemic treatment. **A** Swimmer plot representing therapeutic management and outcome of 23 patients treated with systemic treatment. Each bar represents one subject in the study. **B** Kaplan-Meier curve of PFS in patients treated with first-line dual ICI (*N* = 6 patients) or first-line TKI either alone or combined to ICI (*N* = 10 patients). TKI: anti-angiogenic tyrosine kinase inhibitor. ICI: immune checkpoint inhibitor. PD: progressive disease. KIRC: clear cell renal cell carcinoma. KIRP: papillary renal cell carcinoma. URCC: undifferentiated renal cell carcinoma.

Twenty-three of the 25 patients (92%) received a systemic treatment based on immune checkpoint inhibitors (ICI) and/or anti-angiogenic tyrosine kinase inhibitors (TKI) oriented towards the suspected renal origin, including 17 in the first-line setting. Five patients received RCC-tailored therapy in the second-line setting and one in the third-line setting after previous empiric chemotherapy (Fig. 2A and Supplementary Table 6). The median progression-free survival (mPFS) under the first renal-oriented treatment was 6.3 months (Supplementary Fig. 2A, B). The mPFS of patients treated with TKI as first-line treatment (either alone or combined to ICI) was significantly higher than the mPFS of patients treated with dual ICI in the same setting (12.1 versus 1.5 months, HR: 5.7, 95% CI: 0.97–33.69, *p* = 0.0005) (Fig. 2B). Four patients presenting with initial oligometastatic disease received a local treatment including surgery of lymph node, brain or adrenal metastases, cryotherapy and radiation therapy, with 2 of them being still relapse-free after 33 and 39 months of follow-up. After a median follow-up of 18.5 months, the median overall survival (mOS) of the 25 patients was 52.3 months (Supplementary Fig. 2C, D).

We present a national clinical and biological cohort of rCUP managed in a real-world setting. All diagnoses were prospectively established within our dedicated NatCUPMTB, confirming that integration of multiple parameters including large-scale NGS approaches is feasible in CUPs and can drastically modify the therapeutic strategy^11–13^. Molecular analyses and pathological reviews led to the identification of 7 cases of KIRC, 3 cases of KIRP, 1 case of TFEB-amplified RCC, 1 case of TFE3-rearranged RCC, and 1 case of RMC, all of them showing biological similarities with “classical” RCC, supporting shared cellular origin and molecular oncogenetic processes^14^. Importantly, 12 cases lacked pathological and molecular features specific of dedicated RCC subtypes, despite an over-representation of *NF2* inactivating mutations previously reported in metastatic KIRP^15^. Whether these undifferentiated *NF2*-inactivated rCUP represent a new RCC entity will require additional investigation, however this study suggests that *NF2* inactivation may be a hallmark of a high metastatic potential in RCC. With a mPFS of 6.3 months under the first renal-oriented treatment and a mOS of 52.3 months, this study supports the hypothesis that rCUP constitutes a new entity within the heterogeneous group of CUPs that benefits from tissue-oriented treatment, as suggested in the current international guidelines^2^. This cohort highlights the outmost importance of multidisciplinary expertise for rCUP identification and management. In conclusion, this study provides the first clinical, pathological and molecular description of a cohort of rCUP. Identification of these rare tumors is critical to improve CUPs dismantlement and management.

## Methods

### Patients

NatCUPMTB is a national multidisciplinary tumor board dedicated to CUP patients, established since 2020 and aiming at centralizing clinical data, pathological and molecular analyses to guide diagnosis and treatment. All adult patients discussed at least once during the National CUP MTB between 2020 and 2023 and for which the diagnosis of rCUP was suspected were included in this study. Patients’ characteristics were obtained from referent physicians at the first MTB presentation. Following MTB conclusions and recommendations, therapeutic strategies were the responsibility of the referent physician. Treatment details and survival status were collected prospectively every 2 months or until patients’ death or loss of follow-up. All patients provided written informed consent. The study was conducted in accordance with all relevant ethical regulations including the Declaration of Helsinki.

The study was approved by the NatCUPMTB Institutional regulatory board (Institut Curie DATA220203).

### Samples workflow

After validation of the CUP indication and prescription for WGS, WES and RNAseq analyses in the frame of the National CUP MTB and national initiative Plan France Médecine Genomique 2025, a medical consultation between the referring physician and the patient was organized for the signature of informed consent. Tumor and blood samples were sent to corresponding sites and platforms for extraction of tumor DNA and RNA, as well as constitutional DNA and for sequencing.

### Whole Genome Sequencing, Whole Exome Sequencing and RNA sequencing

Quantification and qualification of nucleic acids were obtained using Spark®, TECAN® and Fragment Analyzer®, Agilent® respectively.

For WGS and WES, fragments were generated by sonication (LE220plus®, Covaris®). Size selection and purification steps were made using magnetic beads (Sera-Mag magnetic beads®, GE Healthcare®). For RNAseq, a cDNA library was generated after poly(A) capture (NEBNext® Poly(A) mRNA Magnetic Isolation Modules, NEBNext® Ultra II RNA First Strand Synthesis Module & Directional RNA Second Strand Module, New England Biolabs®).

Libraries preparations (NEBNext® Ultra II End repair/A-tailing module & Ligation module, New England Biolabs®) were performed without amplification for WGS, and with PCR amplification for WES and RNAseq (KAPA Hifi HotStart ReadyMix, Roche®). Exome capture was done using ‘single-plex’ hybridization (Twist Human Core Exome Kit + IntegraGen Custom v1, Twist BioScience®) and captured regions amplified by PCR (KAPA Hifi HotStart ReadyMix, Roche®).

Final libraries were qualified using capillary migration (Fragment Analyzer®, Agilent®) and quantified by qPCR (NEBNext® Custom 2X Library Quant Kit Master Mix, New England Biolabs® ; QuantStudio 6 Flex Real-Time PCR System®, Life Technologies®). Libraries were sequenced in ‘paired end’ (2×150 cycles for WGS and 2×100 cycles for WES and RNAseq) by SBS technology (Flow Cell S4, NovaSeq 6000®, Illumina®).

### Bioinformatics analyses

Demultiplexing was done from FASTQ files using bcl2fastq®, Illumina® (2.20.0.422). Alignment was performed by bwa_mem-wgs 0.7.15 ; STAR (star 2.7.2d) on the human reference genome (GRCh38.92.fa (GRCh38, release-92, Jul 02, 2018,ftp.ensembl.org).

For WGS and WES, alignment used a Burrows-Wheeler transform (BWAMEM, 0.7.15) and for RNAseq STAR (star 2.7.2d) was used to generate BAM files. An alignment file cleaning process is then performed integrating PCR duplicates for WGS and WES (Picard MarkDuplicates (Picard Tools, 2.8.1)).

Variant calling (SNP and Indels) on constitutional WGS and RNAseq was done using Haplotype Caller (GATK4, v4.1.0.0). Variant calling on WES was done using Mutect 2 (GATK4, v4.1.2.0). Variants annotation were added using SNPeff(4.3t) et SnpSift(4.3t); and the databases of reference were: SNPEff (v4.3t), 1000Genomes (phase3, v2013-05-02), gnomAD exomes (v2.1.1), gnomAD genomes (v3), ClinVar (v20190722), COSMIC (coding, v89), COSMIC (non-coding, v89), dbscSNV (v1.1), dbSNP (v20180418), dbNSFP (v4.0), phastCons (v08-May-2015).

Tumor mutational burden was calculated based on somatic variants (pyTMB (v1.2.0)). Mutational signatures were also extracted (COSMIC database (v3) integrated to SigProfiler (v1.0.9)). MSI status was evaluated from WGS data using MSIsensor2 (v20191121). CNVs were detected by Facet (v0.5.14) and WisecondorX (v1.1.5) and annotated via AnnotSV (v3.0.7) in addition to the following databases: Cytoband (Decembre 2013, USCS), COSMIC (v90). HRD score was also calculated (scarHRD_0.1.1).

Fusion transcripts were analyzed from RNAseq independently using 3 tools : Arriba (v1.2.0), STAR-Fusion (v1.9.0) et FusionCatcher (v1.10); and validated by FusionInspector (STAR-Fusion v1.9.0) followed by annotation using FusionAnnotator (STAR-Fusion, v1.9.0).

RNAseq table counts were generated by Star (v2.7.2d) and Salmon (v1.4.0).

### TransCUPtomics

Transcript per million (tpm) were computed from the raw counts using Gencode v34 to retrieve exon sizes. TransCUPtomics (Vibert 2021) was then used on the tpm values to predict the tissue of origin of each tumor sample. The encoding values from the variational encoder of TransCUPtomics were used to construct the UMAP in two dimensions (R package uwot version 0.1.16).

### Targeted DNA Sequencing

For patients for which no cryopreserved tumor sample was available, targeted DNA sequencing was performed from FFPE samples using a custom NGS panel (marketed by Agilent under the name of SureSelect CD Curie CGP and developed specifically for the molecular analysis of tumors). It is composed of 571 genes of interest in oncology from a diagnosis, prognosis and molecular therapy points of view. The nucleotide sequence (variant calling is performed using Varscan2) as well as the number of copies (deletion and focal amplification) are explored. Briefly, 50 ng of DNA input extracted from FFPE tumors are used to prepare the library with the Agilent SureSelect XT-HS preparation kit according to the manufacturer protocol. Using the design of the 571 genes and an additional backbone of probes across the whole genome with an average resolution of one probe every 200 Kb. This allows us to determine a ploidy and an estimated cellularity, together with a genomic profile spanning every chromosome. The copy number profile for each tumor is estimated using a combination of homemade R scripts and facets package (v0.6.0) with a sex-specific unmatched-germline control previously sequenced using the same panel for normalization. Thirty-two DNA are sequenced per 2×100 Sp flow cell of the NovaSeq Sequencer (Illumina) to reach an average depth of 1,500X and a minimum depth of 100X in the region of interest. The bioinformatics pipeline also includes quality controls metrics determination, identity controls based on polymorphisms, microsatellites instability (MSI) status determination based on MSIsensor2, mutational profiling, and tumor mutational burden (TMB) measurement.

### DNA methylation analyses

Methylation analyses were performed with Infinium MethylationEPIC v2.0 of Illumina. CUP methylation profile were compared to selected data of The Cancer Genome Atlas (TCGA) of metastatic tumors analyzed with Infinium HumanMethylation450K BeadChip. The bioinformatics analysis was done in R (v4.1.2) with the packages minfi (v1.40.0) and wateRmelon (v2.0.0) for importing, quality control and normalizing Illumina DNA methylation arrays data. Only the common probes between both distinct types of arrays (EPIC and 450K) were kept. These probes were filtered to remove localized in sexual chromosome, probes affected by common SNP, and non-specific probes. A detection p-value was returned for every CpG position in each sample. The “m + u” (Methylated + Unmethylated) method was used to compare the total DNA signal for each position to the background signal level. Beta-values were obtained after filters and the top 5000 most variables CpGs by beta-value are kept. The results were plotted with umap (v0.2.10.0) ggplot2 (v3.4.2) R packages.

### Pathological review, immunohistochemistry and in situ hybridization

Cases were reviewed by an expert uropathologist and classified according to the WHO Classification of Urinary and Male Genital Tumors, 5th edition, using a combination of morphological, immunohistochemical and if needed molecular characteristics. All cases where FFPE archival material was available were cut in serial sections of 4 µm thickness and mounted on glass slides. One Hematoxylin Eosin and Saffron staining was performed for each case using Sakura® DRS2000 autostainer. For immunohistochemistry, the following antibodies were used: FH (Santa Cruz Biotechnology, clone J-13, reference SC-100743, dilution 1/200), INI1 BAF47 (BD Biosciences, clone 25/BAF47, reference 612110, dilution 1/50), PAX8 (Zytomed Systems, reference RBK047-06, dilution 1/50), CA IX (Novocastra, clone TH22, reference NCL-L-CAIX, dilution 1/500), MelA (Novocastra, clone A103, reference PA0044, ready to use), TFE3 (BioSB, clone EP285, reference BSB 3226, ready to use), Calretinine (Agilent, clone DAK-Calret 1, reference M7245, dilution 1/100), CK20 (Agilent, clone Ks 20.8, reference M7019, dilution 1/200), Ki67 (Agilent, clone MIB-1, reference GA626, ready to use), BRAF (Abcam, clone VE1, reference AB228461, dilution 1/200), ALK (Cell Signaling, clone D5F3, reference 3633, dilution 1/200), CD163 (Novocastra, clone 10D6, reference NCL-L-CD163, dilution 1/100), CD15 (Agilent, clone Carb-3, reference GA062, ready to use) and CD68 (Agilent, clone KP1, M0814, dilution 1/800).

Antigen retrieval and buffering was done according to validated diagnostic grade protocols on Leica® Bond fully automated staining system, using DAB revelation. Fluorescence in situ Hybridization for TFE3 and TFEB translocation detection were used using break apart probes on Leica® Bond autostainer.

### Statistical and Survival analyses

The categorical data were summarized by the frequencies and percentages, and the continuous covariates were summarized with median, range and numbers of observations. Progression-free survival was defined as the time from new treatment initiation to first evidence of progression or death. Overall survival was defined as the time from diagnosis to death of any cause. Survival curves were plotted using the Kaplan-Meier method and compared using the log-rank test.

### Supplementary information

Supplementary Figures and Tables

## Data Availability

Informed consent signed by the participants does not allow for the data to be deposited into a public or secure access-controlled repository. Targeted DNAseq data are available as supplemental material. Other data including WGS/WES data, RNAseq raw data, raw counts and tpm and DNA methylation b-values will be available from the corresponding authors upon reasonable request and signature of data transfer agreement.
