# Supplementary material for: Metastatic renal cell carcinoma with occult primary: a multicenter prospective cohort
Source: NPJ Precis Oncol. 2024 Jul 18;8:147. doi: 10.1038/s41698-024-00648-0 (PMC11258290; doi:10.1038/s41698-024-00648-0)
Supplement: Supplementary file 1 — Supplementary Figures and Tables [file 41698_2024_648_MOESM1_ESM.pdf]

Supplementary Figure 1: Pathological features of CUPKID samples. A, B: HES (A) and CA9 (B) immuno staining (IHC) of a tumor sample classified as kidney clear cell carcinoma (CUPKID-1). C: HES staining of a representative sample classified as kidney papillary cell carcinoma (CUPKID-10). D: HES staining of CUPKID-3, classified as TFEB-amplified renal cell carcinoma. E, F: HES (E) and INI-1 (F) immunostaining of CUPKID-4, classified as renal medullary carcinoma. G- J: HES (G, I), CA9 (H) and PAX8 (J) immunostaining of CUPKID-20 and CUPKID-8, classified as unclassified renal cell carcinoma.

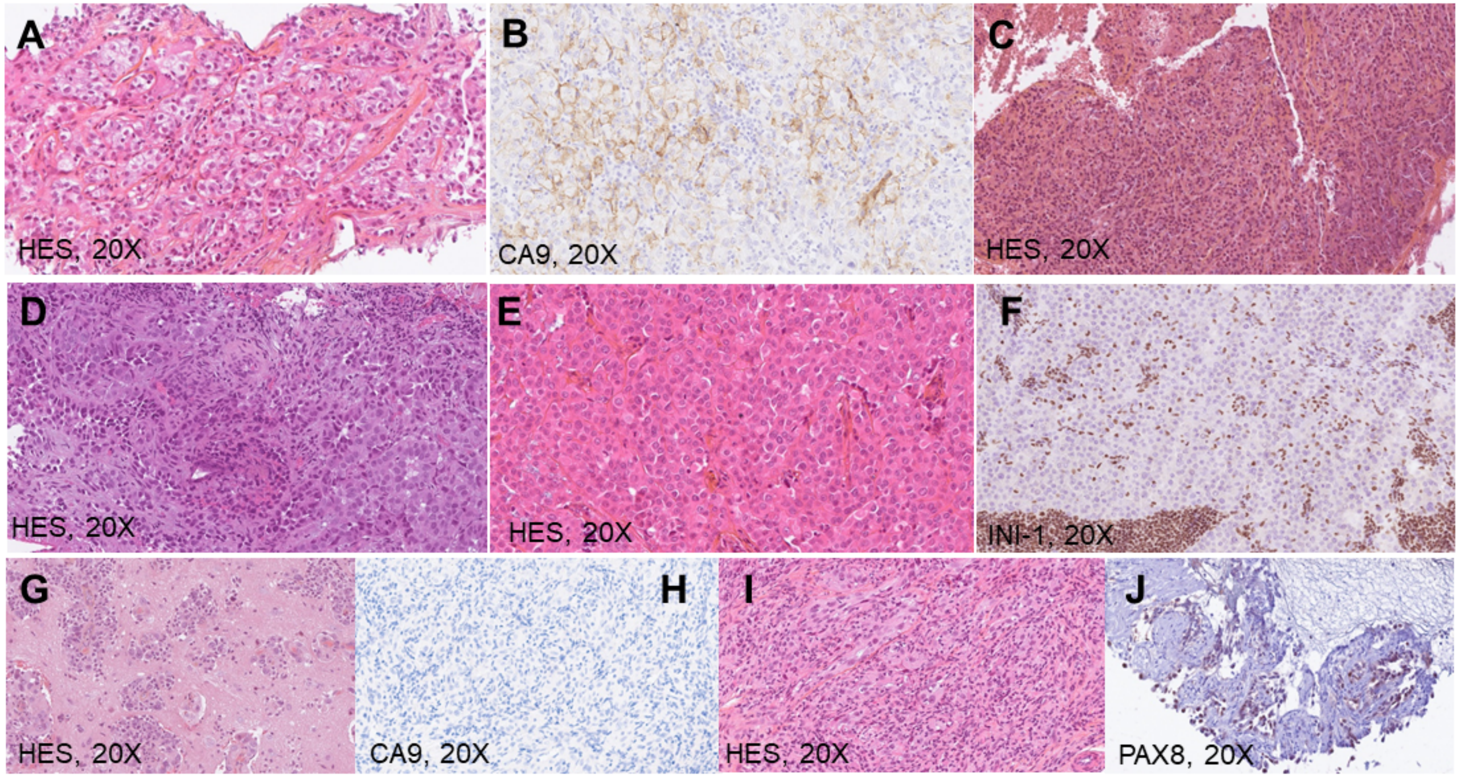

Supplementary Figure 2: Patients survival A: Kaplan-Meier curve of Progression Free Survival (PFS) under the first renal-tailored systemic treatment (N=23 patients). B: Kaplan-Meier curve of Progression Free Survival (PFS) under the first renal-tailored systemic treatment for patients with URCC (N=12 patients) or KIRC (N=6 patients). C: Kaplan-Meir curve of Overall Survival (OS) of the whole cohort (N=25 patients). D: Kaplan-Meir curve of Overall Survival (OS) for patients with URCC (N=12 patients) or KIRC (N=7 patients). Abbreviations: URCC: undifferentiated renal cell carcinoma; KIRC: clear cell renal cell carcinoma.

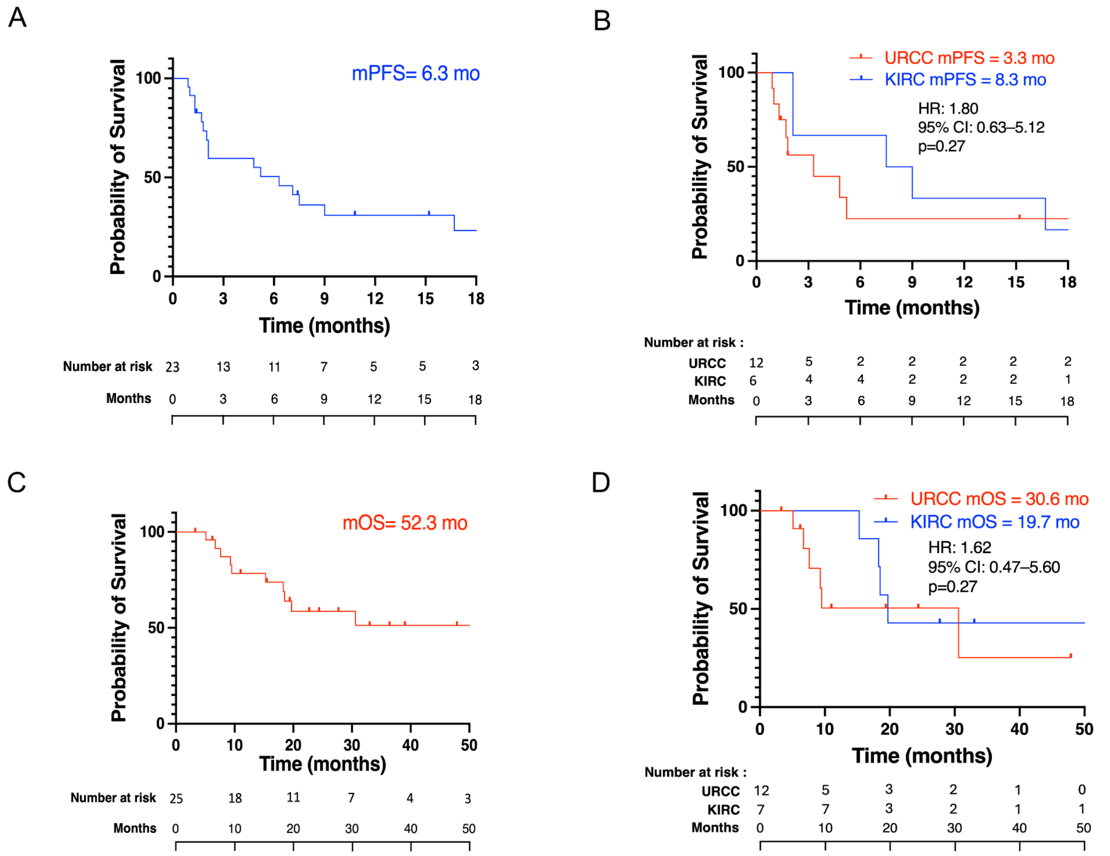

Supplementary Table 1 : Patients' characteristics, pathological review and molecular analyses. Abbreviations: IMDC: International mRCC Database Consortium; WES: Whole Exome Sequencing; WGS: Whole Genome Sequencing; RNAseq: whole transcriptome sequencing.

ID	Gender	Age (years)	IMDC risk score	Metastatic sites at diagnosis	Disease stage at diagnosis	Pathology	Genomics	Methylomics	Transcriptomics
**CUPKID-1**	Female	67	1	Lymph nodes	Loco-regional	Centralized review	WES/WGS	EPIC microarray	RNAseq
**CUPKID-2**	Female	57	1	Bones, adrenal gland	Distant metastases	Local	WES/WGS	EPIC microarray	ND
**CUPKID-3**	Male	58	1	Lymph nodes, adrenal gland	Distant metastases	Centralized review	WES/WGS	EPIC microarray	ND
**CUPKID-4**	Female	29	1	Lymph nodes	Loco-regional	Centralized review	WES/WGS	EPIC microarray	RNAseq
**CUPKID-5**	Male	48	0	Brain	Loco-regional	Centralized review	WES/WGS	EPIC microarray	RNAseq
**CUPKID-6**	Female	85	4	Bones	Distant metastases	Local	DNA panel	EPIC microarray	RNAseq
**CUPKID-7**	Male	60	2	Lymph nodes, bones	Distant metastases	Centralized review	WES/WGS	EPIC microarray	RNAseq
**CUPKID-8**	Male	48	3	Lymph nodes, bones	Distant metastases	Centralized review	ND	ND	RNAseq
**CUPKID-9**	Male	31	3	Lymph nodes, bones	Distant metastases	Centralized review	ND	ND	RNAseq
**CUPKID-10**	Male	73	0	Bones	Loco-regional	Centralized review	ND	ND	ND
**CUPKID-11**	Male	68	3	Lymph nodes, bones, lung, adrenal gland	Distant metastases	Centralized review	ND	ND	ND
**CUPKID-12**	Male	77	2	Bones	Distant metastases	Centralized review	ND	ND	ND
**CUPKID-13**	Female	29	1	Lymph nodes	Distant metastases	Centralized review	ND	ND	ND
**CUPKID-14**	Male	36	1	Lymph nodes	Distant metastases	Local	ND	ND	ND
**CUPKID-15**	Male	45	2	Bones	Distant metastases	Local	ND	ND	ND
**CUPKID-16**	Female	59	0	Bones, adrenal gland	Distant metastases	Centralized review	ND	ND	ND
**CUPKID-17**	Male	43	3	Bones, lymph nodes	Distant metastases	Centralized review	ND	ND	ND

**CUPKID-18**	Male	40	1	Bones, lung	Distant metastases	Centralized review	ND	ND	ND
**CUPKID-19**	Male	45	1	Lymph nodes	Distant metastases	Local	WES/WGS	ND	RNAseq
**CUPKID-20**	Male	68	2	Lymph nodes, bones, lung, thyroid gland	Distant metastases	Centralized review	WES/WGS	ND	RNAseq
**CUPKID-21**	Male	57	1	Lymph nodes, bones, lung	Distant metastases	Local	ND	ND	RNAseq
**CUPKID-22**	Male	70	1	Lymph nodes, bones	Distant metastases	Centralized review	WES/WGS	ND	RNAseq
**CUPKID-23**	Male	64	4	Bones, liver, pleura	Distant metastases	Centralized review	DNA panel	ND	ND
**CUPKID-24**	Female	63	0	Lymph nodes	Loco-regional	Centralized review	DNA panel	ND	ND
**CUPKID-25**	Male	60	2	Lymph nodes	Distant metastases	Local	WES/WGS	ND	RNAseq

Supplementary Table 2: copy number variations identified by targeted DNA sequencing

Barcode	Chr	Start (Hg19)	Stop (Hg19)	Size (Kb)	log2.MedianRatio	Copy	BAF	CancerGene	Ploidy	Cellularity
CUPKID23	chr1	1001177	5697263	4696.086	-1.02448728536755	1	A	TNFRSF14,TP73	2	0.614599193842038
CUPKID23	chr1	6157297	149680266	143522.969	-0.316082337524706	1	A	RPL22,MTOR,MTHFR,SPEN,EPHA2,SDHB,CDA,EPHB2,ID3,SYF2,LDLRAP1,ARID1A,WASF2,LCK,ZMYM4,CSF3R,MYCL,CTPS1,MPL,MUTYH,RAD54L,CDKN2C,JAK1,FUBP1,BCL10,GFI1,RPL5,DPYD,NRAS,CD58,FAM46C,PHGDH,NOTCH2	2	0.614599193842038
CUPKID23	chr1	149753878	246949794	97195.916	0.104769527237086	3	AAB	HIST2H3C,MCL1,SETDB1,RIT1,SDHC,DDR2,ELF3,PIK3C2B,MDM4,H3F3A,ITPKB,FH,AKT3	2	0.614599193842038
CUPKID23	chr1	246950001	248907558	1957.557	-0.185625592109448	2	AA	1_246950001_248907558	2	0.614599193842038
CUPKID23	chr10	235586	135336230	135100.644	-0.0698170476938925	3	AAA	GATA3,BMI1,MYO3A,ABI1,ANKRD26,RET,ARID5B,TET1,FAM213A,PTEN,FAS,TBC1D12,SUFU,NT5C2,SMC3,TCF7L2,PLEKHS1,FGFR2	2	0.614599193842038
CUPKID23	chr11	256715	65206732	64950.017	-0.00926504903124079	3	AAA	HRAS,ZNF143,CAND1.11,MYOD1,FANCF,PAX6,WT1,EXT2,KBTBD4,WDR74,MEN1,DPF2,NEAT1	2	0.614599193842038
CUPKID23	chr11	65206917	65269495	62.578	0.582522390672736	4	AAAA	NEAT1,MALAT1	2	0.614599193842038

CUPKID23	chr11	65269929	134822058	69552.129	-0.110131081078424	2	AA	MALAT1,SF3B2,GSTP1,CCND1,FGF19,FGF4,FGF3,FADD,NUMA1,RNF169,PRKRIR,EMSY,MRE11A,PGR,BIRC3,BIRC2,ATM,SDHD,KMT2A,CBL,CHEK1	2	0.614599193842038
CUPKID23	chr12	164439	56566899	56402.46	-0.0698170476938925	3	AAA	CCND2,FGF6,CHD4,ETV6,CDKN1B,ETNK1,KRAS,ARID2,KMT2D,SMARCD1,ACVR1B,SMUG1,NCKAP1L,ERBB3,SMARCC2	2	0.614599193842038
CUPKID23	chr12	56567693	133830383	77262.69	-0.185625592109448	2	AA	SMARCC2,STAT6,GLI1,CDK4,MDM2,FRS2,PTPRB,NAV3,BTG1,APAF1,TDG,SH2B3,PTPN11,TBX3,HNF1A,BCL7A,NCOR2,EP400,POLE	2	0.614599193842038
CUPKID23	chr13	19748634	109722561	89973.927	-0.185625592109448	2	AA	CDK8,WASF3,FLT3,FLT1,BRCA2,FOXO1,RB1	2	0.614599193842038
CUPKID23	chr13	109722816	111368316	1645.5	0.312643864653254	4	AAAA	IRS2,ING1	2	0.614599193842038
CUPKID23	chr13	111368500	115041325	3672.825	-0.110131081078424	2	AA	ING1	2	0.614599193842038
CUPKID23	chr14	19488974	65544815	46055.841	-0.699428985843242	1	A	CHD8,AJUBA,PRKD1,FOXA1,FANCM,POLE2,OTX2,MAX	2	0.614599193842038

CUPKID23	chr14	65550866	73137890	7587.024	-0.185625592109448	2	AA	MAX,RAD51B,ZFP36L1,DPF3	2	0.614599193842038
CUPKID23	chr14	73138156	107282963	34144.807	-0.699428985843242	1	A	DPF3,COQ6,MLH3,DICER1,BCL11B,TRAF3,XRCC3,AKT1	2	0.614599193842038
CUPKID23	chr15	20488671	22922698	2434.027	1.1960116981888	7	AAAAAAA	15_20488671_22922698	2	0.614599193842038
CUPKID23	chr15	22925605	102376761	79451.156	-0.110131081078424	2	AA	CYFIP1,THBS1,RAD51,MGA,TP53BP1,B2M,TCF12,MAP2K1,SMAD3,CYP1A1,FANCI,IDH2,BLM,CHD2,NR2F2,IGF1R	2	0.614599193842038
CUPKID23	chr16	338085	2125788	1787.703	-0.253424571842556	2	AA	AXIN1,NTHL1,TSC2	2	0.614599193842038
CUPKID23	chr16	2126043	3632774	1506.731	-0.699428985843242	1	A	TSC2,PKD1,SLX4	2	0.614599193842038
CUPKID23	chr16	3633105	15017623	11384.518	0.0582653952744295	3	AAB	SLX4,CREBBP,GRIN2A,CIITA,SOCS1	2	0.614599193842038

CUPKID23	chr16	15018471	18434375	3415.904	-1.02448728536755	1	A	16_15018471_18434375	2	0.614599193842038
CUPKID23	chr16	18437254	85840041	67402.787	-0.0698170476938925	3	AAA	PALB2,RBBP6,ALDOA,SRCAP,BCL7C,PRSS8,BRD7,CHD9,NUP93,CBFB,CTCF,CDH1,NQO1,ZFHX3,PLCG2	2	0.614599193842038
CUPKID23	chr16	85942721	89300182	3357.461	0.686346408522304	5	AAAAA	IRF8	2	0.614599193842038
CUPKID23	chr16	89300320	90241702	941.382	-0.253424571842556	2	AA	FANCA,GAS8-AS1	2	0.614599193842038
CUPKID23	chr17	1070993	25420047	24349.054	-0.699428985843242	1	A	PRPF8,TP53,CHD3,MAP2K4,NCOR1	2	0.614599193842038
CUPKID23	chr17	25420234	81151200	55730.966	-0.110131081078424	2	AA	NF1,SUZ12,RAD51D,MED1,CDK12,ERBB2,IKZF3,SMARCE1,STAT5B,STAT3,BRCA1,KANSL1,HOXB13,ABI3,SPOP,MIR142,RNF43,RAD51C,PPM1D,BRIP1,SMARCD2,CD79B,GNA13,AXIN2,SOX9,H3F3B,SRSF2,RPTOR,ZNF750	2	0.614599193842038
CUPKID23	chr18	252559	46198490	45945.931	-0.253424571842556	2	AA	GATA6,SS18,SETBP1,SLC1A2,SMAD2	2	0.614599193842038

CUPKID23	chr18	46294120	61985464	15691.344	0.104769527237086	3	AAB	SMAD4,TCF4,ALPK2,MALT1,BCL2	2	0.614599193842038
CUPKID23	chr18	61985579	77931131	15945.552	-0.438933215426698	2	AA	18_61985579_77931131	2	0.614599193842038
CUPKID23	chr19	366680	10602180	10235.5	-0.438933215426698	1	A	ELANE,MED16,STK11,APC2,TCF3,GNA11,MAP2K2,CD70,MAP2K7,DNMT1,KEAP1	2	0.614599193842038
CUPKID23	chr19	10602957	15227030	4624.073	-0.253424571842556	2	AA	KEAP1,SMARCA4,CALR	2	0.614599193842038
CUPKID23	chr19	15271388	18266660	2995.272	-0.789339252573334	1	A	NOTCH3,KLF2,JAK3,PIK3R2	2	0.614599193842038
CUPKID23	chr19	18266849	39266238	20999.389	-0.316082337524706	1	A	PIK3R2,MEF2B,CCNE1,CEBPA,KMT2B,DPF1	2	0.614599193842038
CUPKID23	chr19	39266420	59057724	19791.304	-0.571410648295752	1	A	AKT2,AXL,CD79A,CIC,XRCC1,CBLC,ERCC2,PRKD2,ARHGAP35,BCL2L12,POLD1,PPP2R1A,C19MC,U2AF2,ZNF471	2	0.614599193842038

CUPKID23	chr2	83958	103644316	103560.358	-0.185625592109448	2	AA	MYCN,DNMT3A,ASXL2,ALK,ZFP36L2,MSH2,MSH6,FBXO11,FSHR,FANCL,BCL11A,XPO1,PCBP1,TET3	2	0.614599193842038
CUPKID23	chr2	104982460	121743883	16761.423	0.312643864653254	3	AAB	GLI2	2	0.614599193842038
CUPKID23	chr2	121744225	126184908	4440.683	0.76914907094391	5	AAAAA	GLI2	2	0.614599193842038
CUPKID23	chr2	126185124	128741987	2556.863	0.244929073149085	6	AAAAAA	POLR2D	2	0.614599193842038
CUPKID23	chr2	129635464	198265525	68630.061	-0.316082337524706	2	AA	CXCR4,THSD7B,ACVR2A,ACVR1,TBR1,NFE2L2,NCKAP1,PMS1,SF3B1	2	0.614599193842038
CUPKID23	chr2	198265526	198266931	1.405	1.34546633758399	7	AAAAAAA	SF3B1	2	0.814485374863952
CUPKID23	chr2	198266932	241558547	43291.615	-0.316082337524706	2	AA	SF3B1,CASP8,BMPR2,ABI2,CD28,IDH1,ERBB4,BARD1,RQCD1	2	0.614599193842038
CUPKID23	chr20	127539	29611853	29484.314	-0.0698170476938925	3	AAA	PLCB4,FRG1BP	2	0.614599193842038
CUPKID23	chr20	29612241	30161708	549.467	-1.24313242553807	1	A	FRG1BP	2	0.614599193842038
CUPKID23	chr20	30161910	62873991	32712.081	-0.185625592109448	2	AA	BCL2L1,ASXL1,PLCG1,CHD6,PTPRT,AURKA,GNAS,ARFRP1	2	0.614599193842038

CUPKID23	chr21	10996089	48050461	37054.372	-0.511192138686035	1	A	RUNX1,U2AF1	2	0.614599193842038
CUPKID23	chr22	16348213	51072106	34723.893	-0.741047757477362	1	A	CRKL,LZTR1,MAPK1,SMARCB1,MN1,CHEK2,NF2,SOX10,EP300,CYP2D6	2	0.614599193842038
CUPKID23	chr23	2997069	39923896	36926.827	0.0582653952744295	2	AA	FANCB,ZRSR2,EIF1AX,BCOR	2	0.614599193842038
CUPKID23	chr23	39930201	41202398	1272.197	0.76914907094391	2	AA	BCOR,DDX3X	2	0.614599193842038
CUPKID23	chr23	41202618	154348267	113145.649	-0.110131081078424	1	A	DDX3X,KDM6A,RBM10,WAS,GATA1,KDM5C,SMC1A,FOXR2,AMER1,AR,MED12,ZMYM3,TAF1,ATRX,BTK,RAB40A,AGTR2,CUL4B,STAG2,BCORL1,PHF6,BRCC3	2	0.614599193842038

CUPKID23	chr3	274982	55653812	55378.83	-0.571410648295752	1	A	CRBN,FANCD2,BRK1,VHL,RAF1,TGFBR2,MLH1,MYD88,ACVR2B,CTNNB1,ABHD5,SETD2,SMARCC1,RHOA,MST1R,BAP1,PBRM1	2	0.614599193842038
CUPKID23	chr3	57317586	197686322	140368.736	-0.0698170476938925	3	AAA	MITF,FOXP1,ROBO2,ROBO1,LINC02008,EPHA3,CBLB,GATA2,MBD4,EPHB1,STAG1,PIK3CB,FOXL2,RASA2,ATR,ZIC1,MECOM,TERC,PRKCI,TBL1XR1,PIK3CA,ACTL6A,SOX2,KLHL6,BCL6,ZDHHC19	2	0.614599193842038
CUPKID23	chr4	60340	186283186	186222.846	-0.110131081078424	2	AA	FGFR3,WHSC1,SLC2A9,SLIT2,DHX15,UBE2K,PHOX2B,XRCC1,PDGFRA,KIT,KDR,SRP72,EPHA5,DCK,ABCG2,TET2,FAT4,INPP4B,FBXW7,NAF1,DCTD,IRF2,SNX25	2	0.614599193842038
CUPKID23	chr4	186842485	187630590	788.105	0.203940579683492	4	AAAA	FAT1	2	0.614599193842038
CUPKID23	chr4	187630989	190878742	3247.753	-0.185625592109448	2	AA	FAT1	2	0.614599193842038
CUPKID23	chr5	100175	228221	128.046	0.384233316025148	5	AAAAA	SDHA	2	0.614599193842038
CUPKID23	chr5	230835	36460317	36229.482	-0.316082337524706	1	A	SDHA,BRD9,TERT,CTNND2,DROSHA,DNAJC21	2	0.614599193842038
CUPKID23	chr5	36460462	110926210	74465.748	-0.00926504903124079	3	AAA	NIPBL,RICTOR,FGF10,IL6ST,MAP3K1,ERBB2IP,PIK3R1,MSH3,RASA1,MEF2C,CHD1	2	0.614599193842038

CUPKID23	chr5	112043384	138269571	26226.187	0.312643864653254	3	AAB	APC,SNCAIP,RAD50,CDC25C,CTNNA1	2	0.614599193842038
CUPKID23	chr5	140174748	140262168	87.42	1.43168583578215	9	AAAAAAAAA	5_140174748_140262168	2	0.614599193842038
CUPKID23	chr5	140408084	158126181	17718.097	0.312643864653254	3	AAB	ARAP3,CSNK1A1,CSF1R,PDGFRB,FAT2,CYFIP2,EBF1	2	0.614599193842038
CUPKID23	chr5	158126182	158139395	13.213	1.43723444963724	7	AAAAAAA	EBF1	2	0.814485374863952
CUPKID23	chr5	158139396	177683377	19543.981	0.312643864653254	3	AAB	EBF1,NPM1,FGFR4,NSD1,DDX41	2	0.614599193842038
CUPKID23	chr5	177683459	180600292	2916.833	-0.253424571842556	2	AA	FLT4	2	0.614599193842038
CUPKID23	chr6	174798	14190496	14015.698	-0.699428985843242	1	A	IRF4	2	0.614599193842038
CUPKID23	chr6	15649830	27834616	12184.786	0.0582653952744295	3	AAB	HIST1H3B,HIST1H3C,HIST1H1B	2	0.614599193842038
CUPKID23	chr6	27834617	27835272	0.655	1.6411239409032	8	AAAAAAAA	HIST1H1B	2	0.814485374863952
CUPKID23	chr6	27835273	29798248	1962.975	0.0582653952744295	3	AAB	HIST1H1B	2	0.614599193842038
CUPKID23	chr6	29855409	29913343	57.934	0.992068108683844	6	AAAABB	6_29855409_29913343	2	0.614599193842038

CUPKID23	chr6	29913386	170888208	140974.822	-0.0698170476938925	3	AAA	TNF,SLC44A4,NOTCH4,DAXX,FANCE,CDKN1A,PIM1,CCND3,VEGFA,SLC29A1,NFKBIE,PRIM2,BAI3,RIMS1,ZNF292,EPHA7,PRDM1,WASF1,ROS1,SGK1,MYB,BCLAF1,TNFAIP3,GPR126,ESR1,ARID1B,IGF2R,QKI,MLLT4,PHF10	2	0.614599193842038
CUPKID23	chr7	313225	38695431	38382.206	-0.185625592109448	2	AA	CARD11,PMS2,RAC1,BC040327,LOC101927630	2	0.614599193842038
CUPKID23	chr7	39135551	55273031	16137.48	-0.438933215426698	1	A	IKZF1,EGFR	2	0.614599193842038
CUPKID23	chr7	55607128	159099874	103492.746	-0.110131081078424	2	AA	SBDS,BCL7B,CD36,ABCB1,AKAP9,CDK6,SAMD9,SAMD9L,TRRAP,ACTL6B,CUX1,PIK3CG,MET,POT1,SMO,BRAF,EZH2,SMARCD3,RHEB,KMT2C,XRCC2	2	0.614599193842038
CUPKID23	chr8	471805	4475049	4003.244	0.312643864653254	4	AAAA	8_471805_4475049	2	0.614599193842038
CUPKID23	chr8	5616990	47953222	42336.232	-0.789339252573334	1	A	MFHAS1,TMEM66,LEPROTL1,WRN,FGFR1	2	0.614599193842038
CUPKID23	chr8	48486174	146231140	97744.966	-0.00926504903124079	3	AAA	PXDNL,LYN,PREX2,STMN2,NBN,UBR5,RAD21,EXT1,MYC,EPPK1,RECQL4	2	0.614599193842038

CUPKID23	chr9	178294	140642462	140464.168	-0.699428985843242	1	A	SMARCA2,JAK2,CD274,PDCD1LG2,PTPRD,CDKN2A,CDKN2B,TAF1L,FANCG,RMRP,PAX5,GNAQ,FANCC,PTCH1,KLF4,IKBKAP,TLR4,PPP6C,ABL1,NUP214,TSC1,RXRA,NOTCH1,TRAF2	2	0.614599193842038
CUPKID24	chr1	1333598	11319587	9985.989	-0.43355464188806	1	A	TNFRSF14,TP73,RPL22,MTOR	3	0.346509859249058
CUPKID24	chr1	11854476	16265759	4411.283	0.0188521728070398	3	AAA	MTHFR,SPEN	3	0.346509859249058
CUPKID24	chr1	16266013	17380269	1114.256	-0.726920402388448	0		EPHA2,SDHB	3	0.346509859249058
CUPKID24	chr1	17380421	25725155	8344.734	-0.194458251874263	2	AB	SDHB,CDA,EPHB2,ID3,SYF2	3	0.346509859249058
CUPKID24	chr1	25725156	25891497	166.341	-1.0544559657936	0		LDLRAP1	3	0.359732653523833
CUPKID24	chr1	25891498	92944356	67052.858	-0.194458251874263	2	AB	LDLRAP1,ARID1A,WASF2,LCK,ZMYM4,CSF3R,MYCL,CTPS1,MPL,MUTYH,RAD54L,CDKN2C,JAK1,FUBP1,BCL10,GFI1	3	0.346509859249058
CUPKID24	chr1	92944357	92948788	4.431	-1.8796494146736	0		GFI1	3	0.359732653523833
CUPKID24	chr1	92948789	120277370	27328.581	-0.194458251874263	2	AB	GFI1,RPL5,DPYD,NRAS,CD58,FAM46C,PHGDH	3	0.346509859249058

CUPKID24	chr1	120277795	120611836	334.041	-0.43355464188806	1	A	PHGDH,NOTCH2	3	0.346509859249058
CUPKID24	chr1	120658869	149754000	29095.131	-0.0594715624366122	2	AA	1_120658869_149754000	3	0.346509859249058
CUPKID24	chr1	149754001	150551714	797.713	0.64380894376131	8	AAAAAAAA	HIST2H3C,MCL1	3	0.359732653523833
CUPKID24	chr1	150551715	245413472	94861.757	-0.0594715624366122	2	AA	MCL1,SETDB1,RIT1,SDHC,DDR2,ELF3,PIK3C2B,MDM4,H3F3A,ITPKB,FH,AKT3	3	0.346509859249058
CUPKID24	chr1	245413577	248907587	3494.01	-0.262315874821038	1	A	1_245413577_248907587	3	0.346509859249058
CUPKID24	chr10	120052	39011175	38891.123	-0.614559289345979	0		GATA3,BMI1,MYO3A,ABI1,ANKRD26	3	0.346509859249058

CUPKID24	chr10	39012347	42682133	3669.786	5.00566440169114	199	AAAAAAAAAAAAAAAAAAAAAAAAAAAAAAAAAAAAAAAAAAAAAAAAAAAAAAAAAAAAAAAAAAAAAAAAAAAAAAAAAAAAAAAAAAAAAAAAAAAAAAAAAAAAAAAAAAAAAAAAAAAAAAAAAAAAAAAAAAAAAAAAAAAAAAAAAAAAAAAAAAAAAAAAAAAAAAAAAAAAAAAAAAAAAAAAAA	10_39012347_42682133	3	0.346509859249058
CUPKID24	chr10	42682271	104387707	61705.436	-0.262315874821038	1	A	RET,ARID5B,TET1,FAM213A,PTEN,FAS,TBC1D12,SUFU	3	0.346509859249058
CUPKID24	chr10	104387708	104391801	4.093	-1.12320963294372	0		SUFU	3	0.359732653523833

CUPKID24	chr10	104391802	114849037	10457.235	-0.262315874821038	1	A	NT5C2,SMC3,TCF7L2	3	0.346509859249058
CUPKID24	chr10	114849038	114912325	63.287	-0.979889371889906	0		TCF7L2	3	0.359732653523833
CUPKID24	chr10	114912326	135336230	20423.904	-0.262315874821038	1	A	TCF7L2,PLEKHS1,FGFR2	3	0.346509859249058
CUPKID24	chr11	256776	47594369	47337.593	-0.43355464188806	1	A	HRAS,ZNF143,CAND1.11,MYOD1,FANCF,PAX6,WT1,EXT2	3	0.346509859249058
CUPKID24	chr11	47594542	61271371	13676.829	0.199218383850125	3	AAA	KBTBD4	3	0.346509859249058
CUPKID24	chr11	61271372	62609606	1338.234	0.64380894376131	8	AAAAAAAA	WDR74	3	0.359732653523833
CUPKID24	chr11	62609607	63149670	540.063	0.199218383850125	3	AAA	11_62609607_63149670	3	0.346509859249058
CUPKID24	chr11	63718461	65190029	1471.568	-0.262315874821038	2	AA	MEN1,DPF2	3	0.346509859249058
CUPKID24	chr11	65190230	65196411	6.181	0.855156411591979	10	AAAAAAAAAA	NEAT1	3	0.359732653523833
CUPKID24	chr11	65196412	65196821	0.409	0.648556175221939	6	AAAAAA	NEAT1	3	0.346509859249058
CUPKID24	chr11	65196822	65197743	0.921	1.32638263345335	15	AAAAAAAAAAAAAAA	NEAT1	3	0.359732653523833
CUPKID24	chr11	65197744	65197838	0.094	0.648556175221939	6	AAAAAA	NEAT1	3	0.346509859249058
CUPKID24	chr11	65197839	65199013	1.174	0.855156411591979	10	AAAAAAAAAA	NEAT1	3	0.359732653523833

CUPKID24	chr11	65199014	65212880	13.866	0.648556175221939	6	AAAAAA	NEAT1	3	0.346509859249058
CUPKID24	chr11	65212881	65531611	318.73	0.64380894376131	8	AAAAAAAA	NEAT1,MALAT1	3	0.359732653523833
CUPKID24	chr11	65531844	97941076	32409.232	-0.0594715624366122	2	AA	SF3B2,GSTP1,CCND1,FGF19,FGF4,FGF3,FADD,NUMA1,RNF169,PRKRIR,EMSY,MRE11A	3	0.346509859249058
CUPKID24	chr11	97941077	98209605	268.528	-1.34720281767692	0		MRE11A	3	0.359732653523833
CUPKID24	chr11	98209606	134739615	36530.009	-0.0594715624366122	2	AA	MRE11A,PGR,BIRC3,BIRC2,ATM,SDHD,KMT2A,CBL,CHEK1	3	0.346509859249058
CUPKID24	chr12	164460	38919524	38755.064	-0.262315874821038	1	A	CCND2,FGF6,CHD4,ETV6,CDKN1B,ETNK1,KRAS	3	0.346509859249058
CUPKID24	chr12	40364742	40881964	517.222	1.09493025316799	10	AAAAAAAAAA	12_40364742_40881964	3	0.346509859249058
CUPKID24	chr12	40882071	92537809	51655.738	-0.262315874821038	1	A	ARID2,KMT2D,SMARCD1,ACVR1B,SMUG1,NCKAP1L,ERBB3,SMARCC2,STAT6,GLI1,CDK4,MDM2,FRS2,PTPRB,NAV3	3	0.346509859249058
CUPKID24	chr12	92537810	92539344	1.534	0.916689959869796	10	AAAAAAAAAA	BTG1	3	0.359732653523833
CUPKID24	chr12	92539345	121417137	28877.792	-0.262315874821038	1	A	APAF1,TDG,SH2B3,PTPN11,TBX3,HNF1A	3	0.346509859249058
CUPKID24	chr12	121417138	121437114	19.976	-1.12320963294372	0		HNF1A	3	0.359732653523833

CUPKID24	chr12	121437115	124817756	3380.641	-0.262315874821038	1	A	HNF1A,BCL7A,NCOR2	3	0.346509859249058
CUPKID24	chr12	124817944	132549458	7731.514	-0.494729073320595	1	A	NCOR2,EP400	3	0.346509859249058
CUPKID24	chr12	132549459	132589610	40.151	-0.921236571308282	0		EP400	3	0.359732653523833
CUPKID24	chr12	132589611	133830367	1240.756	-0.494729073320595	1	A	POLE	3	0.346509859249058
CUPKID24	chr13	19748709	115041325	95292.616	-0.120698350066416	2	AB	CDK8,WASF3,FLT3,FLT1,BRCA2,FOXO1,RB1,IRS2,ING1	3	0.346509859249058
CUPKID24	chr14	19487495	105411700	85924.205	-0.120698350066416	2	AB	CHD8,AJUBA,PRKD1,FOXA1,FANCM,POLE2,OTX2,MAX,RAD51B,ZFP36L1,DPF3,COQ6,MLH3,DICER1,BCL11B,TRAF3,XRCC3,AKT1	3	0.346509859249058
CUPKID24	chr14	105411835	106126990	715.155	0.123147377023442	3	AAA	14_105411835_106126990	3	0.346509859249058
CUPKID24	chr14	106215309	107245411	1030.102	-0.262315874821038	2	AA	14_106215309_107245411	3	0.346509859249058
CUPKID24	chr15	20488634	102376628	81887.994	-0.315754687250896	1	A	CYFIP1,THBS1,RAD51,MGA,TP53BP1,B2M,TCF12,MAP2K1,SMAD3,CYP1A1,FANCI,IDH2,BLM,CHD2,NR2F2,IGF1R	3	0.346509859249058

CUPKID24	chr16	338161	346946	8.785	-1.18902953953742	0		AXIN1	3	0.359732653523833
CUPKID24	chr16	346947	348416	1.469	-0.315754687250896	1	A	AXIN1	3	0.346509859249058
CUPKID24	chr16	348417	364814	16.397	-1.0544559657936	0		AXIN1	3	0.359732653523833
CUPKID24	chr16	364815	2124553	1759.738	-0.315754687250896	1	A	AXIN1,NTHL1,TSC2	3	0.346509859249058
CUPKID24	chr16	2124554	2136572	12.018	-1.0544559657936	0		TSC2	3	0.359732653523833
CUPKID24	chr16	2136573	2142244	5.671	-0.315754687250896	1	A	TSC2,PKD1	3	0.346509859249058
CUPKID24	chr16	2142245	2142488	0.243	-1.12320963294372	0		PKD1	3	0.359732653523833
CUPKID24	chr16	2142739	2151909	9.17	-1.12320963294372	0		PKD1	3	0.359732653523833
CUPKID24	chr16	2151910	2157078	5.168	-1.14011678409676	0		PKD1	3	0.346509859249058
CUPKID24	chr16	2157079	2169525	12.446	-1.18902953953742	0		PKD1	3	0.359732653523833

CUPKID24	chr16	2185584	3648111	1462.527	0.0188521728070398	3	AAA	PKD1,SLX4	3	0.346509859249058
CUPKID24	chr16	3650990	15023097	11372.107	-0.262315874821038	1	A	SLX4,CREBBP,GRIN2A,CIITA,SOCS1	3	0.346509859249058
CUPKID24	chr16	15024325	18483031	3458.706	-0.682008912445382	0		16_15024325_18483031	3	0.346509859249058
CUPKID24	chr16	18484893	88229771	69744.878	-0.194458251874263	2	AA	PALB2,RBBP6,ALDOA,SRCAP,BCL7C,PRSS8,BRD7,CHD9,NUP93,CBFB,CTCF,CDH1,NQO1,ZFHX3,PLCG2,IRF8	3	0.346509859249058
CUPKID24	chr16	89299280	89825064	525.784	0.0188521728070398	3	AAA	FANCA	3	0.346509859249058
CUPKID24	chr16	89825065	89833576	8.511	0.708438616985323	8	AAAAAAAA	FANCA	3	0.359732653523833
CUPKID24	chr16	89833577	90241702	408.125	0.0188521728070398	3	AAA	FANCA,GAS8-AS1	3	0.346509859249058
CUPKID24	chr17	1070993	6574091	5503.098	-0.194458251874263	2	AA	PRPF8	3	0.346509859249058
CUPKID24	chr17	6574261	25420047	18845.786	-0.494729073320595	1	A	TP53,CHD3,MAP2K4,NCOR1	3	0.346509859249058
CUPKID24	chr17	25420234	73650316	48230.082	-0.194458251874263	2	AA	NF1,SUZ12,RAD51D,MED1,CDK12,ERBB2,IKZF3,SMARCE1,STAT5B,STAT3,BRCA1,KANSL1,HOXB13,ABI3,SPOP,MIR142,RNF43,RAD51C,PPM1D,BRIP1,SMARCD2,CD79B,GNA13,AXIN2,SOX9	3	0.346509859249058
CUPKID24	chr17	73650485	78829209	5178.724	0.123147377023442	3	AAB	H3F3B,SRSF2,RPTOR	3	0.346509859249058

CUPKID24	chr17	78829424	79323711	494.287	-0.43355464188806	1	A	RPTOR	3	0.346509859249058
CUPKID24	chr17	79441086	81151311	1710.225	0.356812250171223	4	AAAA	ZNF750	3	0.346509859249058
CUPKID24	chr18	73444	18520278	18446.834	0.199218383850125	4	AAAB	18_73444_18520278	3	0.346509859249058
CUPKID24	chr18	18541476	52946988	34405.512	-0.194458251874263	2	AB	GATA6,SS18,SETBP1,SLC1A2,SMAD2,SMAD4,TCF4	3	0.346509859249058
CUPKID24	chr18	52969617	78012849	25043.232	0.0188521728070398	3	AAB	TCF4,ALPK2,MALT1,BCL2	3	0.346509859249058
CUPKID24	chr19	366774	855860	489.086	-0.876297498946652	0		ELANE	3	0.359732653523833
CUPKID24	chr19	855861	11170308	10314.447	-0.554250418809112	1	A	ELANE,MED16,STK11,APC2,TCF3,GNA11,MAP2K2,CD70,MAP2K7,DNMT1,KEAP1,SMARCA4	3	0.346509859249058
CUPKID24	chr19	11170309	11173129	2.82	-0.876297498946652	0		SMARCA4	3	0.359732653523833
CUPKID24	chr19	11173130	14682606	3509.476	-0.554250418809112	1	A	CALR	3	0.346509859249058
CUPKID24	chr19	14682607	15303497	620.89	-0.921236571308282	0		NOTCH3	3	0.359732653523833

CUPKID24	chr19	15303498	18245335	2941.837	-0.554250418809112	1	A	NOTCH3,KLF2,JAK3	3	0.346509859249058
CUPKID24	chr19	18245527	40739864	22494.337	-0.262315874821038	1	A	PIK3R2,MEF2B,CCNE1,CEBPA,KMT2B,DPF1,AKT2	3	0.346509859249058
CUPKID24	chr19	40739865	40747798	7.933	-0.979889371889906	0		AKT2	3	0.359732653523833
CUPKID24	chr19	40747799	57027673	16279.874	-0.262315874821038	1	A	AKT2,AXL,CD79A,CIC,XRCC1,CBLC,ERCC2,PRKD2,ARHGAP35,BCL2L12,POLD1,PPP2R1A,C19MC,U2AF2,ZNF471	3	0.346509859249058
CUPKID24	chr19	57029938	59057872	2027.934	-0.614559289345979	0		ZNF471	3	0.346509859249058
CUPKID24	chr2	83989	133009109	132925.12	-0.194458251874263	2	AA	MYCN,DNMT3A,ASXL2,ALK,ZFP36L2,MSH2,MSH6,FBXO11,FSHR,FANCL,BCL11A,XPO1,PCBP1,TET3,GLI2,POLR2D	3	0.346509859249058
CUPKID24	chr2	133012743	133039156	26.413	1.14155518808979	10	AAAAAAAAAA	2_133012743_133039156	3	0.346509859249058
CUPKID24	chr2	133208949	241558547	108349.598	-0.262315874821038	1	A	CXCR4,THSD7B,ACVR2A,ACVR1,TBR1,NFE2L2,NCKAP1,PMS1,SF3B1,CASP8,BMPR2,ABI2,CD28,IDH1,ERBB4,BARD1,RQCD1	3	0.346509859249058

CUPKID24	chr20	127493	13789061	13661.568	-0.194458251874263	2	AB	PLCB4	3	0.346509859249058
CUPKID24	chr20	13973987	32783487	18809.5	0.0188521728070398	3	AAA	FRG1BP,BCL2L1,ASXL1	3	0.346509859249058
CUPKID24	chr20	33018008	35179496	2161.488	0.0188521728070398	3	AAA	20_33018008_35179496	3	0.346509859249058
CUPKID24	chr20	35179649	41408956	6229.307	-0.494729073320595	1	A	PLCG1,CHD6,PTPRT	3	0.346509859249058
CUPKID24	chr20	41419916	62873991	21454.075	-0.194458251874263	2	AB	PTPRT,AURKA,GNAS,ARFRP1	3	0.346509859249058
CUPKID24	chr21	9826194	36164310	26338.116	-0.120698350066416	2	AB	21_9826194_36164310	3	0.346509859249058
CUPKID24	chr21	36164311	36194041	29.73	-1.0544559657936	0		RUNX1	3	0.359732653523833
CUPKID24	chr21	36194042	48050461	11856.419	-0.120698350066416	2	AB	RUNX1,U2AF1	3	0.346509859249058
CUPKID24	chr22	16349307	24125891	7776.584	-0.120698350066416	2	AB	CRKL,LZTR1,MAPK1	3	0.346509859249058
CUPKID24	chr22	24125892	24129129	3.237	0.64380894376131	8	AAAAAAAA	SMARCB1	3	0.359732653523833

CUPKID24	chr22	24129130	51071999	26942.869	-0.120698350066416	2	AB	SMARCB1,MN1,CHEK2,NF2,SOX10,EP300,CYP2D6	3	0.346509859249058
CUPKID24	chr23	2997069	154522512	151525.443	-0.262315874821038	1	A	FANCB,ZRSR2,EIF1AX,BCOR,DDX3X,KDM6A,RBM10,WAS,GATA1,KDM5C,SMC1A,FOXR2,AMER1,AR,MED12,ZMYM3,TAF1,ATRX,BTK,RAB40A,AGTR2,CUL4B,STAG2,BCORL1,PHF6,BRCC3	3	0.346509859249058
CUPKID24	chr3	275101	78667227	78392.126	-0.120698350066416	2	AB	CRBN,FANCD2,BRK1,VHL,RAF1,TGFBR2,MLH1,MYD88,ACVR2B,CTNNB1,ABHD5,SETD2,SMARCC1,RHOA,MST1R,BAP1,PBRM1,MITF,FOXP1,ROBO2,ROBO1	3	0.346509859249058
CUPKID24	chr3	78667228	78683236	16.008	-0.876297498946652	0		ROBO1	3	0.359732653523833
CUPKID24	chr3	78683237	195051895	116368.658	-0.120698350066416	2	AB	ROBO1,LINC02008,EPHA3,CBLB,GATA2,MBD4,EPHB1,STAG1,PIK3CB,FOXL2,RASA2,ATR,ZIC1,MECOM,TERC,PRKCI,TBL1XR1,PIK3CA,ACTL6A,SOX2,KLHL6,BCL6	3	0.346509859249058
CUPKID24	chr3	195384754	195704217	319.463	-1.14011678409676	0		3_195384754_195704217	3	0.346509859249058
CUPKID24	chr3	195706589	195891822	185.233	0.123147377023442	3	AAA	3_195706589_195891822	3	0.346509859249058
CUPKID24	chr3	195891823	195892513	0.69	2.38598918244156	34	AAAAAAAAAAAAAAAAAAAAAAAAAAAAAAAAAA	ZDHHC19	3	0.359732653523833

CUPKID24	chr3	195892514	197686215	1793.701	0.123147377023442	3	AAA	3_195892514_197686215	3	0.346509859249058
CUPKID24	chr4	60483	1806519	1746.036	-1.40081985086022	0		FGFR3	3	0.359732653523833
CUPKID24	chr4	1806520	1957150	150.63	-0.43355464188806	1	A	FGFR3,WHSC1	3	0.346509859249058
CUPKID24	chr4	1957151	1976530	19.379	-0.921236571308282	0		WHSC1	3	0.359732653523833
CUPKID24	chr4	1976531	182479223	180502.692	-0.43355464188806	1	A	WHSC1,SLC2A9,SLIT2,DHX15,UBE2K,PHOX2B,XRCC1,PDGFRA,KIT,KDR,SRP72,EPHA5,DCK,ABCG2,TET2,FAT4,INPP4B,FBXW7,NAF1	3	0.346509859249058
CUPKID24	chr4	182479224	183824040	1344.816	-1.18902953953742	0		DCTD	3	0.359732653523833
CUPKID24	chr4	183824041	190878674	7054.633	-0.43355464188806	1	A	DCTD,IRF2,SNX25,FAT1	3	0.346509859249058
CUPKID24	chr5	100175	31410756	31310.581	-0.194458251874263	2	AA	SDHA,BRD9,TERT,CTNND2,DROSHA	3	0.346509859249058
CUPKID24	chr5	31410923	42984581	11573.658	0.123147377023442	3	AAA	DROSHA,DNAJC21,NIPBL,RICTOR	3	0.346509859249058
CUPKID24	chr5	42984713	141059569	98074.856	-0.262315874821038	1	A	FGF10,IL6ST,MAP3K1,ERBB2IP,PIK3R1,MSH3,RASA1,MEF2C,CHD1,APC,SNCAIP,RAD50,CDC25C,CTNNA1,ARAP3	3	0.346509859249058

CUPKID24	chr5	141059570	141836763	777.193	0.64380894376131	8	AAAAAAAA	ARAP3	3	0.359732653523833
CUPKID24	chr5	141836764	149500767	7664.003	-0.262315874821038	1	A	CSNK1A1,CSF1R,PDGFRB	3	0.346509859249058
CUPKID24	chr5	149500997	156721986	7220.989	-0.374946640846489	1	A	PDGFRB,FAT2,CYFIP2	3	0.346509859249058
CUPKID24	chr5	156723675	177683459	20959.784	-0.120698350066416	2	AB	CYFIP2,EBF1,NPM1,FGFR4,NSD1,DDX41	3	0.346509859249058
CUPKID24	chr5	178604215	180600297	1996.082	-0.682008912445382	0		FLT4	3	0.346509859249058
CUPKID24	chr6	174798	21120558	20945.76	0.0188521728070398	3	AAA	IRF4	3	0.346509859249058
CUPKID24	chr6	22202679	29795636	7592.957	0.488982861538231	5	AAAAA	HIST1H3B,HIST1H3C,HIST1H1B	3	0.346509859249058
CUPKID24	chr6	29795768	29797553	1.785	-0.194458251874263	2	AA	6_29795768_29797553	3	0.346509859249058
CUPKID24	chr6	29797696	31430891	1633.195	0.563707840623296	6	AAAABB	6_29797696_31430891	3	0.346509859249058
CUPKID24	chr6	31431082	32191844	760.762	0.0188521728070398	3	AAB	TNF,SLC44A4,NOTCH4	3	0.346509859249058
CUPKID24	chr6	32441396	32729467	288.071	0.488982861538231	5	AAABB	6_32441396_32729467	3	0.346509859249058

CUPKID24	chr6	32729468	33397671	668.203	0.776465548879126	9	AAAAAAAAA	DAXX	3	0.359732653523833
CUPKID24	chr6	33397672	38845314	5447.642	0.488982861538231	5	AAABB	FANCE,CDKN1A,PIM1	3	0.346509859249058
CUPKID24	chr6	38845550	44191856	5346.306	0.0188521728070398	3	AAB	CCND3,VEGFA,SLC29A1	3	0.346509859249058
CUPKID24	chr6	44191857	44201807	9.95	1.26562584618494	14	AAAAAAAAAAAAAA	SLC29A1	3	0.359732653523833
CUPKID24	chr6	44201808	44230239	28.431	0.0188521728070398	3	AAB	SLC29A1,NFKBIE	3	0.346509859249058
CUPKID24	chr6	44230240	44233488	3.248	0.64380894376131	8	AAAAAAAA	NFKBIE	3	0.359732653523833
CUPKID24	chr6	44233489	141664366	97430.877	0.0188521728070398	3	AAB	NFKBIE,PRIM2,BAI3,RIMS1,ZNF292,EPHA7,PRDM1,WASF1,ROS1,SGK1,MYB,BCLAF1,TNFAIP3	3	0.346509859249058
CUPKID24	chr6	142421775	170888285	28466.51	-0.494729073320595	1	A	GPR126,ESR1,ARID1B,IGF2R,QKI,MLLT4,PHF10	3	0.346509859249058
CUPKID24	chr7	313264	6022629	5709.365	-0.726920402388448	0		CARD11,PMS2	3	0.346509859249058
CUPKID24	chr7	6026530	54137586	48111.056	-0.120698350066416	2	AB	PMS2,RAC1,BC040327,LOC101927630,IKZF1	3	0.346509859249058
CUPKID24	chr7	54137675	55260615	1122.94	-0.614559289345979	0		EGFR	3	0.346509859249058

CUPKID24	chr7	55266456	97535232	42268.776	-0.0594715624366122	2	AA	EGFR,SBDS,BCL7B,CD36,ABCB1,AKAP9,CDK6,SAMD9,SAMD9L	3	0.346509859249058
CUPKID24	chr7	97904377	101917520	4013.143	-0.43355464188806	1	A	TRRAP,ACTL6B,CUX1	3	0.346509859249058
CUPKID24	chr7	101917521	101925383	7.862	-1.0544559657936	0		CUX1	3	0.359732653523833
CUPKID24	chr7	101925384	106507954	4582.57	-0.43355464188806	1	A	CUX1	3	0.346509859249058
CUPKID24	chr7	106508264	153161300	46653.036	-0.0594715624366122	2	AA	PIK3CG,MET,POT1,SMO,BRAF,EZH2,SMARCD3,RHEB,KMT2C,XRCC2	3	0.346509859249058
CUPKID24	chr7	153161433	159099912	5938.479	-0.262315874821038	2	AA	7_153161433_159099912	3	0.346509859249058
CUPKID24	chr8	471805	90990478	90518.673	-0.262315874821038	1	A	MFHAS1,TMEM66,LEPROTL1,WRN,FGFR1,PXDNL,LYN,PREX2,STMN2,NBN	3	0.346509859249058
CUPKID24	chr8	90990479	90996748	6.269	0.64380894376131	8	AAAAAAAA	NBN	3	0.359732653523833
CUPKID24	chr8	90996749	146231140	55234.391	-0.262315874821038	1	A	NBN,UBR5,RAD21,EXT1,MYC,EPPK1,RECQL4	3	0.346509859249058

CUPKID24	chr9	178431	5080717	4902.286	-0.554250418809112	1	A	SMARCA2,JAK2	3	0.346509859249058
CUPKID24	chr9	5080718	5291111	210.393	-0.921236571308282	0		JAK2	3	0.359732653523833
CUPKID24	chr9	5291112	7356056	2064.944	-0.554250418809112	1	A	CD274,PDCD1LG2	3	0.346509859249058
CUPKID24	chr9	7499180	19246290	11747.11	0.123147377023442	3	AAA	PTPRD	3	0.346509859249058
CUPKID24	chr9	19246443	27605798	8359.355	-0.819918855788977	0		CDKN2A,CDKN2B	3	0.346509859249058
CUPKID24	chr9	27605979	79623442	52017.463	0.0188521728070398	3	AAA	TAF1L,FANCG,RMRP,PAX5	3	0.346509859249058
CUPKID24	chr9	79623537	98242427	18618.89	-0.682008912445382	0		GNAQ,FANCC,PTCH1	3	0.346509859249058
CUPKID24	chr9	98242925	133033504	34790.579	-0.43355464188806	1	A	PTCH1,KLF4,IKBKAP,TLR4,PPP6C	3	0.346509859249058
CUPKID24	chr9	133033505	134001008	967.503	-0.876297498946652	0		ABL1	3	0.359732653523833
CUPKID24	chr9	134001009	137313410	3312.401	-0.43355464188806	1	A	NUP214,TSC1,RXRA	3	0.346509859249058

CUPKID24	chr9	137313725	137313741	0.016	-0.969965510840395	0		9_137313725_137313741	3	0.346509859249058
CUPKID24	chr9	137313742	139412911	2099.169	-1.23291501745957	0		RXRA,NOTCH1	3	0.359732653523833
CUPKID24	chr9	139412912	141044632	1631.72	-0.969965510840395	0		NOTCH1,TRAF2	3	0.346509859249058
CUPKID6	chr1	1333598	16247523	14913.925	-0.574563892944901	1	A	TNFRSF14,TP73,RPL22,MTOR,MTHFR,SPEN	2	0.697508202365211
CUPKID6	chr1	16247524	16262926	15.402	-1.05512284131289	0		SPEN	2	NA
CUPKID6	chr1	16262927	120457850	104194.923	-0.574563892944901	1	A	SPEN,EPHA2,SDHB,CDA,EPHB2,ID3,SYF2,LDLRAP1,ARID1A,WASF2,LCK,ZMYM4,CSF3R,MYCL,CTPS1,MPL,MUTYH,RAD54L,CDKN2C,JAK1,FUBP1,BCL10,GFI1,RPL5,DPYD,NRAS,CD58,FAM46C,PHGDH	2	0.697508202365211
CUPKID6	chr1	120458004	248907587	128449.583	0.0516961741241415	2	AB	NOTCH2,HIST2H3C,MCL1,SETDB1,RIT1,SDHC,DDR2,ELF3,PIK3C2B,MDM4,H3F3A,ITPKB,FH,AKT3	2	0.697508202365211

CUPKID6	chr2	83989	44536716	44452.727	0.121204366785636	2	AB	MYCN,DNMT3A,ASXL2,ALK,ZFP36L2	2	0.697508202365211
CUPKID6	chr2	46511939	58468569	11956.63	-0.140293576639265	2	AA	MSH2,MSH6,FBXO11,FSHR,FANCL	2	0.697508202365211
CUPKID6	chr2	59854481	133009109	73154.628	0.121204366785636	2	AB	BCL11A,XPO1,PCBP1,TET3,GLI2,POLR2D	2	0.697508202365211
CUPKID6	chr2	133012743	133209142	196.399	0.591683006329309	5	AAAAA	2_133012743_133209142	2	0.697508202365211
CUPKID6	chr2	133619580	241558547	107938.967	-0.0100917328864911	2	AB	CXCR4,THSD7B,ACVR2A,ACVR1,TBR1,NFE2L2,NCKAP1,PMS1,SF3B1,CASP8,BMPR2,ABI2,CD28,IDH1,ERBB4,BARD1,RQCD1	2	0.697508202365211
CUPKID6	chr3	275101	87521026	87245.925	-0.574563892944901	1	A	CRBN,FANCD2,BRK1,VHL,RAF1,TGFBR2,MLH1,MYD88,ACVR2B,CTNNB1,ABHD5,SETD2,SMARCC1,RHOA,MST1R,BAP1,PBRM1,MITF,FOXP1,ROBO2,ROBO1,LINC02008	2	0.697508202365211
CUPKID6	chr3	88145924	193033547	104887.623	-0.0100917328864911	2	AB	EPHA3,CBLB,GATA2,MBD4,EPHB1,STAG1,PIK3CB,FOXL2,RASA2,ATR,ZIC1,MECOM,TERC,PRKCI,TBL1XR1,PIK3CA,ACTL6A,SOX2,KLHL6,BCL6	2	0.697508202365211
CUPKID6	chr3	193343761	195717290	2373.529	0.121204366785636	2	AA	3_193343761_195717290	2	0.697508202365211
CUPKID6	chr3	195891908	197686215	1794.307	0.0516961741241415	2	AA	ZDHHC19	2	0.697508202365211

CUPKID6	chr4	60483	48956187	48895.704	-0.51296104987317	1	A	FGFR3,WHSC1,SLC2A9,SLIT2,DHX15,UBE2K,PHOX2B,XRCC1	2	0.697508202365211
CUPKID6	chr4	48956374	92808323	43851.949	-0.0100917328864911	2	AB	PDGFRA,KIT,KDR,SRP72,EPHA5,DCK,ABCG2	2	0.697508202365211
CUPKID6	chr4	94793323	138005330	43212.007	-0.574563892944901	1	A	TET2,FAT4	2	0.697508202365211
CUPKID6	chr4	138005525	151020770	13015.245	-0.199512144151601	2	AA	INPP4B	2	0.697508202365211
CUPKID6	chr4	151970152	190878674	38908.522	-0.574563892944901	1	A	FBXW7,NAF1,DCTD,IRF2,SNX25,FAT1	2	0.697508202365211
CUPKID6	chr5	100175	34441016	34340.841	0.121204366785636	2	AB	SDHA,BRD9,TERT,CTNND2,DROSHA	2	0.697508202365211
CUPKID6	chr5	34662418	79950707	45288.289	-0.0100917328864911	2	AB	DNAJC21,NIPBL,RICTOR,FGF10,IL6ST,MAP3K1,ERBB2IP,PIK3R1,MSH3	2	0.697508202365211
CUPKID6	chr5	79950708	79950823	0.115	-1.19022455060357	0		MSH3	2	NA
CUPKID6	chr5	79950824	85998660	6047.836	-0.0100917328864911	2	AB	MSH3	2	0.697508202365211

CUPKID6	chr5	86564890	86686576	121.686	-0.574563892944901	1	A	RASA1	2	0.697508202365211
CUPKID6	chr5	88025138	140721446	52696.308	-0.0100917328864911	2	AB	MEF2C,CHD1,APC,SNCAIP,RAD50,CDC25C,CTNNA1	2	0.697508202365211
CUPKID6	chr5	141033476	141059569	26.093	0.121204366785636	2	AB	ARAP3	2	0.697508202365211
CUPKID6	chr5	141059570	141141361	81.791	2.2636425188657	12	AAAAAAAAAAAA	ARAP3	2	NA
CUPKID6	chr5	141141362	156738830	15597.468	0.121204366785636	2	AB	CSNK1A1,CSF1R,PDGFRB,FAT2,CYFIP2	2	0.697508202365211
CUPKID6	chr5	156741238	176722219	19980.981	-0.0100917328864911	2	AA	CYFIP2,EBF1,NPM1,FGFR4,NSD1	2	0.697508202365211
CUPKID6	chr5	176938695	180600297	3661.602	0.253648611363494	3	AAB	DDX41,FLT4	2	0.697508202365211
CUPKID6	chr6	174798	29855849	29681.051	-0.0100917328864911	2	AB	IRF4,HIST1H3B,HIST1H3C,HIST1H1B	2	0.697508202365211
CUPKID6	chr6	29855952	33287504	3431.552	0.0516961741241415	2	AB	TNF,SLC44A4,NOTCH4,DAXX	2	0.697508202365211
CUPKID6	chr6	33288075	170888285	137600.21	0.0516961741241415	2	AB	DAXX,FANCE,CDKN1A,PIM1,CCND3,VEGFA,SLC29A1,NFKBIE,PRIM2,BAI3,RIMS1,ZNF292,EPHA7,PRDM1,WASF1,ROS1,SGK1,MYB,BCLAF1,TNFAIP3,GPR126,ESR1,ARID1B,IGF2R,QKI,MLLT4,PHF10	2	0.697508202365211

CUPKID6	chr7	313259	91116723	90803.464	0.0516961741241415	2	AB	CARD11,PMS2,RAC1,BC040327,LOC101927630,IKZF1,EGFR,SBDS,BCL7B,CD36,ABCB1	2	0.697508202365211
CUPKID6	chr7	91403147	91727487	324.34	-0.271023729773355	1	A	AKAP9	2	0.697508202365211
CUPKID6	chr7	91729127	151879590	60150.463	0.0516961741241415	2	AB	AKAP9,CDK6,SAMD9,SAMD9L,TRRAP,ACTL6B,CUX1,PIK3CG,MET,POT1,SMO,BRAF,EZH2,SMARCD3,RHEB,KMT2C	2	0.697508202365211
CUPKID6	chr7	151880296	152372994	492.698	-0.199512144151601	2	AA	KMT2C,XRCC2	2	0.697508202365211
CUPKID6	chr7	152373252	159099912	6726.66	0.121204366785636	2	AA	7_152373252_159099912	2	0.697508202365211
CUPKID6	chr8	471886	30916880	30444.994	-0.364417383145274	1	A	MFHAS1,TMEM66,LEPROTL1,WRN	2	0.697508202365211
CUPKID6	chr8	30921773	32997219	2075.446	-0.748273915784421	1	A	WRN	2	0.697508202365211
CUPKID6	chr8	34079703	51846792	17767.089	0.190063835139882	2	AA	FGFR1	2	0.697508202365211
CUPKID6	chr8	52232577	131444902	79212.325	-0.0100917328864911	2	AB	PXDNL,LYN,PREX2,STMN2,NBN,UBR5,RAD21,EXT1,MYC	2	0.697508202365211

CUPKID6	chr8	131679540	146231140	14551.6	0.190063835139882	3	AAB	EPPK1,RECQL4	2	0.697508202365211
CUPKID6	chr9	178431	21971288	21792.857	-0.574563892944901	1	A	SMARCA2,JAK2,CD274,PDCD1LG2,PTPRD,CDKN2A	2	0.697508202365211
CUPKID6	chr9	21971289	22009040	37.751	-1.60264036099152	0		CDKN2A,CDKN2B	2	NA
CUPKID6	chr9	22009041	141044565	119035.524	-0.574563892944901	1	A	TAF1L,FANCG,RMRP,PAX5,GNAQ,FANCC,PTCH1,KLF4,IKBKAP,TLR4,PPP6C,ABL1,NUP214,TSC1,RXRA,NOTCH1,TRAF2	2	0.697508202365211
CUPKID6	chr10	119952	32105570	31985.618	-0.651343985582907	1	A	GATA3,BMI1,MYO3A,ABI1,ANKRD26	2	0.697508202365211
CUPKID6	chr10	33307191	39012347	5705.156	-0.140293576639265	2	AA	10_33307191_39012347	2	0.697508202365211
CUPKID6	chr10	39128867	42599912	3471.045	4.78412105268782	76	AAAAAAAAAAAAAAAAAAAAAAAAAAAAAAAAAAAAAAAAAAAAAAAAAAAAAAAAAAAAAAAAAAAAAAAAAAAA	10_39128867_42599912	2	0.697508202365211
CUPKID6	chr10	42660136	54173096	11512.96	-0.199512144151601	2	AA	RET	2	0.697508202365211

CUPKID6	chr10	54854441	135336230	80481.789	0.0516961741241415	2	AB	ARID5B,TET1,FAM213A,PTEN,FAS,TBC1D12,SUFU,NT5C2,SMC3,TCF7L2,PLEKHS1,FGFR2	2	0.697508202365211
CUPKID6	chr11	256714	1051715	795.001	-0.140293576639265	2	AA	HRAS	2	0.697508202365211
CUPKID6	chr11	1606129	2526617	920.488	0.84648659907846	5	AAAAA	11_1606129_2526617	2	0.697508202365211
CUPKID6	chr11	2526691	65211940	62685.249	0.0516961741241415	2	AB	ZNF143,CAND1.11,MYOD1,FANCF,PAX6,WT1,EXT2,KBTBD4,WDR74,MEN1,DPF2,NEAT1	2	0.697508202365211
CUPKID6	chr11	65211979	65827073	615.094	-0.199512144151601	2	AA	NEAT1,MALAT1,SF3B2	2	0.697508202365211
CUPKID6	chr11	65827224	105519832	39692.608	0.0516961741241415	2	AB	SF3B2,GSTP1,CCND1,FGF19,FGF4,FGF3,FADD,NUMA1,RNF169,PRKRIR,EMSY,MRE11A,PGR,BIRC3,BIRC2	2	0.697508202365211
CUPKID6	chr11	105519887	124034823	18514.936	-0.574563892944901	1	A	ATM,SDHD,KMT2A,CBL	2	0.697508202365211
CUPKID6	chr11	124078705	124135669	56.964	-0.092649704200844	2	AA	11_124078705_124135669	2	0.697508202365211
CUPKID6	chr11	125490759	134739615	9248.856	-0.574563892944901	1	A	CHEK1	2	0.697508202365211

CUPKID6	chr12	164460	38543869	38379.409	0.0516961741241415	2	AB	CCND2,FGF6,CHD4,ETV6,CDKN1B,ETNK1,KRAS	2	0.697508202365211
CUPKID6	chr12	38600655	40944102	2343.447	1.38900388801357	6	AAAAAA	12_38600655_40944102	2	0.697508202365211
CUPKID6	chr12	40944140	133830367	92886.227	0.0516961741241415	2	AB	ARID2,KMT2D,SMARCD1,ACVR1B,SMUG1,NCKAP1L,ERBB3,SMARCC2,STAT6,GLI1,CDK4,MDM2,FRS2,PTPRB,NAV3,BTG1,APAF1,TDG,SH2B3,PTPN11,TBX3,HNF1A,BCL7A,NCOR2,EP400,POLE	2	0.697508202365211
CUPKID6	chr13	19748709	26928056	7179.347	-0.414139623091936	1	A	CDK8	2	0.697508202365211
CUPKID6	chr13	26956913	32972884	6015.971	-0.703858260037993	1	A	CDK8,WASF3,FLT3,FLT1,BRCA2	2	0.697508202365211
CUPKID6	chr13	33051261	43835306	10784.045	-0.315158605648812	1	A	FOXO1	2	0.697508202365211
CUPKID6	chr13	45169705	56843525	11673.82	-0.748273915784421	1	A	RB1	2	0.697508202365211
CUPKID6	chr13	58308015	115041325	56733.31	-0.455360505069198	1	A	IRS2,ING1	2	0.697508202365211
CUPKID6	chr14	19487495	20265401	777.906	-1.78580636634778	0		14_19487495_20265401	2	0.697508202365211

CUPKID6	chr14	20677546	45606290	24928.744	0.0516961741241415	2	AB	CHD8,AJUBA,PRKD1,FOXA1,FANCM	2	0.697508202365211
CUPKID6	chr14	45609786	103369836	57760.05	-0.0100917328864911	2	AB	FANCM,POLE2,OTX2,MAX,RAD51B,ZFP36L1,DPF3,COQ6,MLH3,DICER1,BCL11B,TRAF3	2	0.697508202365211
CUPKID6	chr14	103371638	105415224	2043.586	0.190063835139882	3	AAB	TRAF3,XRCC3,AKT1	2	0.697508202365211
CUPKID6	chr14	105415279	105418166	2.887	-1.00341254861969	1	A	14_105415279_105418166	2	0.697508202365211
CUPKID6	chr14	105418293	107245435	1827.142	0.190063835139882	2	AA	14_105418293_107245435	2	0.697508202365211
CUPKID6	chr15	20488634	102376628	81887.994	-0.574563892944901	1	A	CYFIP1,THBS1,RAD51,MGA,TP53BP1,B2M,TCF12,MAP2K1,SMAD3,CYP1A1,FANCI,IDH2,BLM,CHD2,NR2F2,IGF1R	2	0.697508202365211
CUPKID6	chr16	338161	16452036	16113.875	0.0516961741241415	2	AB	AXIN1,NTHL1,TSC2,PKD1,SLX4,CREBBP,GRIN2A,CIITA,SOCS1	2	0.697508202365211
CUPKID6	chr16	16452744	17214305	761.561	-0.703858260037993	1	A	16_16452744_17214305	2	0.697508202365211
CUPKID6	chr16	17214477	18487498	1273.021	0.980795211609724	6	AAAAAA	16_17214477_18487498	2	0.697508202365211

CUPKID6	chr16	18487927	67671828	49183.901	-0.0100917328864911	2	AB	PALB2,RBBP6,ALDOA,SRCAP,BCL7C,PRSS8,BRD7,CHD9,NUP93,CBFB,CTCF	2	0.697508202365211
CUPKID6	chr16	68486912	88229771	19742.859	0.121204366785636	2	AB	CDH1,NQO1,ZFHX3,PLCG2,IRF8	2	0.697508202365211
CUPKID6	chr16	89299245	89300250	1.005	1.22661182369049	6	AAAAAA	16_89299245_89300250	2	0.697508202365211
CUPKID6	chr16	89546990	90241702	694.712	-0.0100917328864911	2	AB	FANCA,GAS8-AS1	2	0.697508202365211
CUPKID6	chr17	1071061	81151311	80080.25	0.0516961741241415	2	AB	PRPF8,TP53,CHD3,MAP2K4,NCOR1,NF1,SUZ12,RAD51D,MED1,CDK12,ERBB2,IKZF3,SMARCE1,STAT5B,STAT3,BRCA1,KANSL1,HOXB13,ABI3,SPOP,MIR142,RNF43,RAD51C,PPM1D,BRIP1,SMARCD2,CD79B,GNA13,AXIN2,SOX9,H3F3B,SRSF2,RPTOR,ZNF750	2	0.697508202365211
CUPKID6	chr18	73444	15182692	15109.248	0.0516961741241415	2	AB	18_73444_15182692	2	0.697508202365211
CUPKID6	chr18	18516158	18519318	3.16	3.85931582441682	40	AAAAAAAAAAAAAAAAAAAAAAAAAAAAAAAAAAAAAAAA	18_18516158_18519318	2	0.697508202365211
CUPKID6	chr18	18520278	24595872	6075.594	0.0516961741241415	2	AB	GATA6,SS18	2	0.697508202365211
CUPKID6	chr18	24687324	78012849	53325.525	-0.574563892944901	1	A	SETBP1,SLC1A2,SMAD2,SMAD4,TCF4,ALPK2,MALT1,BCL2	2	0.697508202365211

CUPKID6	chr19	366696	3119101	2752.405	-0.574563892944901	1	A	ELANE,MED16,STK11,APC2,TCF3,GNA11	2	0.697508202365211
CUPKID6	chr19	3119102	4095645	976.543	-1.19022455060357	0		MAP2K2	2	NA
CUPKID6	chr19	4095646	28138223	24042.577	-0.574563892944901	1	A	MAP2K2,CD70,MAP2K7,DNMT1,KEAP1,SMARCA4,CALR,NOTCH3,KLF2,JAK3,PIK3R2,MEF2B	2	0.697508202365211
CUPKID6	chr19	28417356	29661730	1244.374	0.0516961741241415	2	AA	19_28417356_29661730	2	0.697508202365211
CUPKID6	chr19	29912457	45995432	16082.975	-0.455360505069198	1	A	CCNE1,CEBPA,KMT2B,DPF1,AKT2,AXL,CD79A,CIC,XRCC1,CBLC,ERCC2	2	0.697508202365211
CUPKID6	chr19	46375608	59057872	12682.264	0.0516961741241415	2	AB	PRKD2,ARHGAP35,BCL2L12,POLD1,PPP2R1A,C19MC,U2AF2,ZNF471	2	0.697508202365211
CUPKID6	chr20	127493	7885184	7757.691	0.121204366785636	2	AA	20_127493_7885184	2	0.697508202365211
CUPKID6	chr20	8120724	9438164	1317.44	-0.0100917328864911	2	AA	PLCB4	2	0.697508202365211
CUPKID6	chr20	9440331	29611909	20171.578	0.121204366785636	2	AB	PLCB4,FRG1BP	2	0.697508202365211
CUPKID6	chr20	29612241	30336655	724.414	-0.199512144151601	2	AA	FRG1BP,BCL2L1	2	0.697508202365211

CUPKID6	chr20	30372114	62873991	32501.877	0.0516961741241415	2	AB	ASXL1,PLCG1,CHD6,PTPRT,AURKA,GNAS,ARFRP1	2	0.697508202365211
CUPKID6	chr21	9826194	48050461	38224.267	0.121204366785636	2	AB	RUNX1,U2AF1	2	0.697508202365211
CUPKID6	chr22	16349307	51071999	34722.692	-0.51296104987317	1	A	CRKL,LZTR1,MAPK1,SMARCB1,MN1,CHEK2,NF2,SOX10,EP300,CYP2D6	2	0.697508202365211
CUPKID6	chr23	2997069	154522512	151525.443	0.0516961741241415	2	AB	FANCB,ZRSR2,EIF1AX,BCOR,DDX3X,KDM6A,RBM10,WAS,GATA1,KDM5C,SMC1A,FOXR2,AMER1,AR,MED12,ZMYM3,TAF1,ATRX,BTK,RAB40A,AGTR2,CUL4B,STAG2,BCORL1,PHF6,BRCC3	2	0.697508202365211

<u>Supplementary Table 3: Samples from the TCGA database and from Masaouel *et al*. used as controls for DNA methylation analyses.</u>

	CUPKID-ID	Data source	TCGA case submitter ID	Histological type	Stage (M)	Stage N	Stage T	Stage TNM
**1**	TCGA-KIRP-1	TCGA Database	TCGA-G7-6789	KIRP	MX	N2	T3a	Stage IV
**2**	TCGA-KIRP-2	TCGA Database	TCGA-G7-A8LB	KIRP	MX	NX	T2a	Stage IV
**3**	TCGA-KIRP-3	TCGA Database	TCGA-4A-A93X	KIRP	MX	N1	T3a	Stage IV
**4**	TCGA-KIRP-4	TCGA Database	TCGA-G7-6793	KIRP	MX	N2	T3a	Stage IV
**5**	TCGA-KIRP-5	TCGA Database	TCGA-GL-6846	KIRP	MX	N1	T4	Stage IV
**6**	TCGA-KIRP-6	TCGA Database	TCGA-A4-A57E	KIRP	M1	N0	T2a	Stage IV
**7**	TCGA-KIRP-7	TCGA Database	TCGA-BQ-5893	KIRP	M1	N1	T3a	Stage IV
**8**	TCGA-KIRP-8	TCGA Database	TCGA-SX-A7SM	KIRP	M1	N1	T3a	Stage IV
**9**	TCGA-KIRP-9	TCGA Database	TCGA-2Z-A9J7	KIRP	M1	NX	T2	Stage IV
**10**	TCGA-KIRP-10	TCGA Database	TCGA-F9-A8NY	KIRP	M1	N1	T4	Stage IV
**11**	TCGA-KIRP-11	TCGA Database	TCGA-BQ-5877	KIRP	M1	N1	T3a	Stage IV
**12**	TCGA-KIRP-12	TCGA Database	TCGA-BQ-5889	KIRP	M1	N1	T3b	Stage IV
**13**	TCGA-KIRP-13	TCGA Database	TCGA-BQ-5894	KIRP	M1	N1	T3b	Stage IV
**14**	TCGA-KIRP-14	TCGA Database	TCGA-AL-7173	KIRP	M0	N2	T3	Stage IV
**15**	TCGA-KIRP-15	TCGA Database	TCGA-GL-A59R	KIRP	MX	N0	T3c	Stage III
**16**	TCGA-KIRP-16	TCGA Database	TCGA-F9-A4JJ	KIRP	MX	NX	T3a	Stage III
**17**	TCGA-KIRP-17	TCGA Database	TCGA-IA-A40Y	KIRP	MX	N1	T3a	Stage III
**18**	TCGA-KIRP-18	TCGA Database	TCGA-B1-A47M	KIRP	MX	NX	T3a	Stage III
**19**	TCGA-KIRP-19	TCGA Database	TCGA-Y8-A896	KIRP	MX	NX	T3a	Stage III
**20**	TCGA-KIRP-20	TCGA Database	TCGA-IA-A40U	KIRP	MX	N0	T3b	Stage III
**21**	TCGA-KIRP-21	TCGA Database	TCGA-Q2-A5QZ	KIRP	MX	NX	T3a	Stage III
**22**	TCGA-KIRP-22	TCGA Database	TCGA-G7-A8LD	KIRP	MX	N1	T3a	Stage III
**23**	TCGA-KIRP-23	TCGA Database	TCGA-2Z-A9JI	KIRP	MX	NX	T3a	Stage III
**24**	TCGA-KIRP-24	TCGA Database	TCGA-A4-A7UZ	KIRP	MX	N1	T3a	Stage III
**25**	TCGA-KIRP-25	TCGA Database	TCGA-P4-AAVL	KIRP	MX	N0	T3b	Stage III

**26**	TCGA-KIRP-26	TCGA Database	TCGA-G7-6797	KIRP	MX	NX	T1a	Stage III
**27**	TCGA-KIRP-27	TCGA Database	TCGA-P4-AAVK	KIRP	MX	N1	T3a	Stage III
**28**	TCGA-KIRP-28	TCGA Database	TCGA-UZ-A9PQ	KIRP	MX	N1	T2	Stage III
**29**	TCGA-KIRP-29	TCGA Database	TCGA-A4-A5Y1	KIRP	MX	N1	T1b	Stage III
**30**	TCGA-KIRP-30	TCGA Database	TCGA-P4-A5EA	KIRP	MX	N1	T3a	Stage III
**31**	TCGA-KIRC-1	TCGA Database	TCGA-CJ-4923	KIRC	M1	NX	T3a	Stage IV
**32**	TCGA-KIRC-2	TCGA Database	TCGA-B0-4688	KIRC	M1	N0	T4	Stage IV
**33**	TCGA-KIRC-3	TCGA Database	TCGA-B0-4690	KIRC	M1	N0	T4	Stage IV
**34**	TCGA-KIRC-4	TCGA Database	TCGA-B0-4691	KIRC	M1	N0	T2	Stage IV
**35**	TCGA-KIRC-5	TCGA Database	TCGA-B0-4697	KIRC	M1	NX	T3b	Stage IV
**36**	TCGA-KIRC-6	TCGA Database	TCGA-B0-4700	KIRC	M1	NX	T4	Stage IV
**37**	TCGA-KIRC-7	TCGA Database	TCGA-B0-4701	KIRC	M1	N0	T3a	Stage IV
**38**	TCGA-KIRC-8	TCGA Database	TCGA-B0-4703	KIRC	M1	N0	T3a	Stage IV
**39**	TCGA-KIRC-9	TCGA Database	TCGA-B0-4712	KIRC	M1	NX	T3a	Stage IV
**40**	TCGA-KIRC-10	TCGA Database	TCGA-B0-4714	KIRC	M1	NX	T3b	Stage IV
**41**	TCGA-KIRC-11	TCGA Database	TCGA-B0-4814	KIRC	M1	N0	T4	Stage IV
**42**	TCGA-KIRC-12	TCGA Database	TCGA-B0-4819	KIRC	M1	NX	T3b	Stage IV
**43**	TCGA-KIRC-13	TCGA Database	TCGA-B0-4828	KIRC	M1	NX	T2	Stage IV
**44**	TCGA-KIRC-14	TCGA Database	TCGA-B0-4841	KIRC	M1	NX	T2	Stage IV
**45**	TCGA-KIRC-15	TCGA Database	TCGA-B0-4844	KIRC	M1	NX	T3a	Stage IV
**46**	TCGA-KIRC-16	TCGA Database	TCGA-B0-4845	KIRC	M1	NX	T3a	Stage IV
**47**	TCGA-KIRC-17	TCGA Database	TCGA-B0-4846	KIRC	M1	N0	T3a	Stage IV
**48**	TCGA-KIRC-18	TCGA Database	TCGA-B0-4847	KIRC	M1	NX	T3a	Stage IV
**49**	TCGA-KIRC-19	TCGA Database	TCGA-B0-5080	KIRC	M1	N0	T3a	Stage IV
**50**	TCGA-KIRC-20	TCGA Database	TCGA-B0-5092	KIRC	M1	N0	T1a	Stage IV
**51**	TCGA-KIRC-21	TCGA Database	TCGA-B0-5094	KIRC	M1	N0	T3b	Stage IV
**52**	TCGA-KIRC-22	TCGA Database	TCGA-B0-5107	KIRC	M1	N0	T2	Stage IV
**53**	TCGA-KIRC-23	TCGA Database	TCGA-B0-5115	KIRC	M1	N0	T2	Stage IV
**54**	TCGA-KIRC-24	TCGA Database	TCGA-B0-5712	KIRC	M1	N0	T2	Stage IV

**55**	TCGA-KIRC-25	TCGA Database	TCGA-B2-5639	KIRC	M1	NX	T3	Stage IV
**56**	TCGA-KIRC-26	TCGA Database	TCGA-B4-5377	KIRC	M1	N0	T3	Stage IV
**57**	TCGA-KIRC-27	TCGA Database	TCGA-B8-4622	KIRC	M1	N0	T3a	Stage IV
**58**	TCGA-KIRC-28	TCGA Database	TCGA-BP-5178	KIRC	M1	NX	T3a	Stage IV
**59**	TCGA-KIRC-29	TCGA Database	TCGA-BP-5201	KIRC	M1	N0	T3b	Stage IV
**60**	TCGA-KICH-1	TCGA Database	TCGA-KN-8426	KICH	M1	NX	T3a	Stage IV
**61**	TCGA-KICH-2	TCGA Database	TCGA-KL-8339	KICH	M1	N1	T3a	Stage IV
**62**	TCGA-KICH-3	TCGA Database	TCGA-KL-8336	KICH	M0	N2	T3b	Stage IV
**63**	TCGA-KICH-4	TCGA Database	TCGA-KL-8341	KICH	M0	N2	T3b	Stage IV
**64**	TCGA-KICH-5	TCGA Database	TCGA-KM-8440	KICH	MX	NX	T3a	Stage III
**65**	TCGA-KICH-6	TCGA Database	TCGA-KN-8433	KICH	'--	NX	T3a	Stage III
**66**	TCGA-KICH-7	TCGA Database	TCGA-KO-8408	KICH	'--	N1	T3a	Stage III
**67**	TCGA-KICH-8	TCGA Database	TCGA-KN-8429	KICH	'--	N0	T3a	Stage III
**68**	TCGA-KICH-9	TCGA Database	TCGA-KO-8405	KICH	M0	N0	T3a	Stage III
**69**	TCGA-KICH-10	TCGA Database	TCGA-KO-8416	KICH	M0	N0	T3a	Stage III
**70**	TCGA-KICH-11	TCGA Database	TCGA-KL-8344	KICH	M0	N0	T3a	Stage III
**71**	TCGA-KICH-12	TCGA Database	TCGA-KL-8345	KICH	M0	N0	T3a	Stage III
**72**	TCGA-KICH-13	TCGA Database	TCGA-KL-8326	KICH	M0	N0	T3a	Stage III
**73**	TCGA-LUAD-1	TCGA Database	TCGA-93-A4JP	Lung adenocarcinoma	M1b	NX	TX	Stage IV
**74**	TCGA-LUAD-2	TCGA Database	TCGA-55-8094	Lung adenocarcinoma	M1b	N0	T2b	Stage IV
**75**	TCGA-LUAD-3	TCGA Database	TCGA-55-8512	Lung adenocarcinoma	M1b	N1	T1a	Stage IV
**76**	TCGA-LUAD-4	TCGA Database	TCGA-L9-A5IP	Lung adenocarcinoma	M1b	N2	T3	Stage IV
**77**	TCGA-LUAD-5	TCGA Database	TCGA-55-8620	Lung adenocarcinoma	M1b	N1	T1a	Stage IV
**78**	TCGA-LUAD-6	TCGA Database	TCGA-97-8171	Lung adenocarcinoma	M1a	N2	T2a	Stage IV
**79**	TCGA-LUAD-7	TCGA Database	TCGA-93-A4JN	Lung adenocarcinoma	M1a	N0	T2a	Stage IV
**80**	TCGA-LUAD-8	TCGA Database	TCGA-78-7145	Lung adenocarcinoma	M1	N1	T4	Stage IV
**81**	TCGA-LUAD-9	TCGA Database	TCGA-53-7624	Lung adenocarcinoma	M1	N0	T2	Stage IV
**82**	TCGA-LUAD-10	TCGA Database	TCGA-78-7156	Lung adenocarcinoma	M1	N1	T4	Stage IV

**83**	TCGA-LUSC-1	TCGA Database	TCGA-NC-A5HF	Squamous-cell adenocarcinoma	MX	N0	T4	Stage IIIB
**84**	TCGA-LUSC-2	TCGA Database	TCGA-77-8145	Squamous-cell adenocarcinoma	M0	N1	T4	Stage IIIB
**85**	TCGA-LUSC-3	TCGA Database	TCGA-NK-A7XE	Squamous-cell adenocarcinoma	M0	N2	T4	Stage IIIB
**86**	TCGA-LUSC-4	TCGA Database	TCGA-77-7335	Squamous-cell adenocarcinoma	M0	N2	T4	Stage IIIB
**87**	TCGA-LUSC-5	TCGA Database	TCGA-37-A5EN	Squamous-cell adenocarcinoma	M0	N2	T4	Stage IIIB
**88**	TCGA-LUSC-6	TCGA Database	TCGA-NC-A5HP	Squamous-cell adenocarcinoma	M1b	N0	T2a	Stage IV
**89**	TCGA-LUSC-7	TCGA Database	TCGA-34-8455	Squamous-cell adenocarcinoma	M1a	N0	T4	Stage IV
**90**	TCGA-LUSC-8	TCGA Database	TCGA-58-8386	Squamous-cell adenocarcinoma	M1	NX	T3	Stage IV
**91**	TCGA-LUSC-9	TCGA Database	TCGA-18-3417	Squamous-cell adenocarcinoma	M1	N1	T2	Stage IV
**92**	TCGA-PAAD-1	TCGA Database	TCGA-YY-A8LH	Pancreatic adenocarcinoma	MX	N1	T3	Stage IIB
**93**	TCGA-PAAD-2	TCGA Database	TCGA-FZ-5926	Pancreatic adenocarcinoma	M0	N1	T3	Stage III
**94**	TCGA-PAAD-3	TCGA Database	TCGA-IB-8126	Pancreatic adenocarcinoma	M0	N1	T4	Stage III
**95**	TCGA-PAAD-4	TCGA Database	TCGA-2L-AAQJ	Pancreatic adenocarcinoma	MX	N0	T4	Stage III
**96**	TCGA-PAAD-5	TCGA Database	TCGA-HZ-8001	Pancreatic adenocarcinoma	MX	N0	T4	Stage III
**97**	TCGA-PAAD-6	TCGA Database	TCGA-FZ-5923	Pancreatic adenocarcinoma	M1	N1	T4	Stage IV
**98**	TCGA-PAAD-7	TCGA Database	TCGA-HZ-8636	Pancreatic adenocarcinoma	M1	N0	T3	Stage IV
**99**	TCGA-PAAD-8	TCGA Database	TCGA-HZ-A9TJ	Pancreatic adenocarcinoma	M1	N0	T3	Stage IV
**100**	TCGA-PAAD-9	TCGA Database	TCGA-IB-7644	Pancreatic adenocarcinoma	M1	N1	T3	Stage IV
**101**	TCGA-CHOL-1	TCGA Database	TCGA-3X-AAVA	Cholangiocarcinoma	M0	NX	T2b	Stage II
**102**	TCGA-CHOL-2	TCGA Database	TCGA-WD-A7RX	Cholangiocarcinoma	MX	NX	T2b	Stage II
**103**	TCGA-CHOL-3	TCGA Database	TCGA-YR-A95A	Cholangiocarcinoma	M1	NX	T2	Stage IV
**104**	TCGA-CHOL-4	TCGA Database	TCGA-W5-AA36	Cholangiocarcinoma	M1	N0	T3	Stage IV
**105**	TCGA-CHOL-5	TCGA Database	TCGA-W5-AA2W	Cholangiocarcinoma	M0	N1	T2a	Stage IVA
**106**	TCGA-CHOL-6	TCGA Database	TCGA-ZH-A8Y1	Cholangiocarcinoma	M0	N1	T3	Stage IVA

**107**	TCGA-CHOL-7	TCGA Database	TCGA-3X-AAVB	Cholangiocarcinoma	M1	N1	T3	Stage IVB
**108**	TCGA-CHOL-8	TCGA Database	TCGA-W5-AA2X	Cholangiocarcinoma	M1	N1	T2b	Stage IVB
**109**	TCGA-CHOL-9	TCGA Database	TCGA-ZH-A8Y5	Cholangiocarcinoma	M1	N1	T3	Stage IVB
**110**	TCGA-COAD-1	TCGA Database	TCGA-F4-6809	Colon adenocarcinoma	M1	N1	T3	Stage IVA
**111**	TCGA-COAD-2	TCGA Database	TCGA-CM-5868	Colon adenocarcinoma	M1a	N1a	T4a	Stage IVA
**112**	TCGA-COAD-3	TCGA Database	TCGA-CM-4747	Colon adenocarcinoma	M1a	N1b	T4a	Stage IVA
**113**	TCGA-COAD-4	TCGA Database	TCGA-A6-6648	Colon adenocarcinoma	M1a	N0	T3	Stage IVA
**114**	TCGA-COAD-5	TCGA Database	TCGA-NH-A50U	Colon adenocarcinoma	M1a	N0	T4a	Stage IVA
**115**	TCGA-COAD-6	TCGA Database	TCGA-CM-5862	Colon adenocarcinoma	M1a	N1a	T3	Stage IVA
**116**	TCGA-COAD-7	TCGA Database	TCGA-A6-6142	Colon adenocarcinoma	M1a	N1a	T3	Stage IVA
**117**	TCGA-COAD-8	TCGA Database	TCGA-CM-6678	Colon adenocarcinoma	M1a	N1c	T4a	Stage IVA
**118**	TCGA-COAD-9	TCGA Database	TCGA-NH-A6GC	Colon adenocarcinoma	M1b	N1b	T4b	Stage IVB
**119**	TCGA-COAD-10	TCGA Database	TCGA-CM-6675	Colon adenocarcinoma	M1b	N2b	T3	Stage IVB
**120**	TCGA-MESO-1	TCGA Database	TCGA-SC-AA5Z	Mesothelioma	M0	N2	T4	Stage IV
**121**	TCGA-MESO-2	TCGA Database	TCGA-LK-A4NW	Mesothelioma	M0	N2	T4	Stage IV
**122**	TCGA-MESO-3	TCGA Database	TCGA-YS-A95B	Mesothelioma	M0	N3	T4	Stage IV
**123**	TCGA-MESO-4	TCGA Database	TCGA-3U-A98H	Mesothelioma	M0	N2	T4	Stage IV
**124**	TCGA-MESO-5	TCGA Database	TCGA-YS-AA4M	Mesothelioma	M1	N0	T1b	Stage IV
**125**	TCGA-MESO-6	TCGA Database	TCGA-SH-A7BD	Mesothelioma	M1	N0	T2	Stage IV
**126**	TCGA-MESO-7	TCGA Database	TCGA-ZN-A9VO	Mesothelioma	M1	N2	T4	Stage IV
**127**	TCGA-MESO-8	TCGA Database	TCGA-TS-A7OY	Mesothelioma	MX	N3	T3	Stage IV
**128**	TCGA-MESO-9	TCGA Database	TCGA-TS-A7OU	Mesothelioma	MX	N0	T4	Stage IV
**129**	TCGA-MESO-10	TCGA Database	TCGA-TS-A8AI	Mesothelioma	MX	N1	T4	Stage IV
**130**	TCGA-STAD-1	TCGA Database	TCGA-CG-5722	Stomach adenocarcinoma	M1	N2	T3	Stage IV
**131**	TCGA-STAD-2	TCGA Database	TCGA-IN-A7NR	Stomach adenocarcinoma	M1	N3	T3	Stage IV
**132**	TCGA-STAD-3	TCGA Database	TCGA-CG-5730	Stomach adenocarcinoma	M1	N1	T2	Stage IV
**133**	TCGA-STAD-4	TCGA Database	TCGA-CG-5724	Stomach adenocarcinoma	M1	N3	T3	Stage IV

**134**	TCGA-STAD-5	TCGA Database	TCGA-VQ-A8PJ	Stomach adenocarcinoma	M1	N2	T4	Stage IV
**135**	TCGA-STAD-6	TCGA Database	TCGA-BR-7957	Stomach adenocarcinoma	M1	N3a	T3	Stage IV
**136**	TCGA-STAD-7	TCGA Database	TCGA-R5-A804	Stomach adenocarcinoma	M1	N2	T3	Stage IV
**137**	TCGA-STAD-8	TCGA Database	TCGA-BR-A453	Stomach adenocarcinoma	M1	N3a	T4a	Stage IV
**138**	TCGA-STAD-9	TCGA Database	TCGA-BR-7196	Stomach adenocarcinoma	M1	N3a	T3	Stage IV
**139**	TCGA-STAD-10	TCGA Database	TCGA-CG-5719	Stomach adenocarcinoma	M1	N0	T4	Stage IV
**140**	TCGA-BLCA-1	TCGA Database	TCGA-C4-A0EZ	Bladder Urothelial Carcinoma	M1	N1	T3a	Stage IV
**141**	TCGA-BLCA-2	TCGA Database	TCGA-FD-A3SL	Bladder Urothelial Carcinoma	M1	N2	T4a	Stage IV
**142**	TCGA-BLCA-3	TCGA Database	TCGA-FD-A3SM	Bladder Urothelial Carcinoma	M1	N2	T3a	Stage IV
**143**	TCGA-BLCA-4	TCGA Database	TCGA-FD-A6TF	Bladder Urothelial Carcinoma	M1	N2	T3b	Stage IV
**144**	TCGA-BLCA-5	TCGA Database	TCGA-YC-A9TC	Bladder Urothelial Carcinoma	M1	x	x	Stage IV
**145**	TCGA-BLCA-6	TCGA Database	TCGA-4Z-AA84	Bladder Urothelial Carcinoma	M1	N2	T3a	Stage IV
**146**	TCGA-BLCA-7	TCGA Database	TCGA-FJ-A3ZE	Bladder Urothelial Carcinoma	M1	N3	x	Stage IV
**147**	TCGA-BLCA-8	TCGA Database	TCGA-H4-A2HQ	Bladder Urothelial Carcinoma	M1	NX	x	Stage IV
**148**	TCGA-BLCA-9	TCGA Database	TCGA-KQ-A41P	Bladder Urothelial Carcinoma	M1	N3	T3b	Stage IV
**149**	TCGA-BLCA-10	TCGA Database	TCGA-ZF-AA5N	Bladder Urothelial Carcinoma	M1	NX	T2	Stage IV
**150**	NCBI-GEO-DATA-1	GEO Database : GSE211942	NA	Renal Medullary Carcinoma	x	x	x	x
**151**	NCBI-GEO-DATA-2	GEO Database : GSE211942	NA	Renal Medullary Carcinoma	x	x	x	x
**152**	NCBI-GEO-DATA-3	GEO Database : GSE211942	NA	Renal Medullary Carcinoma	x	x	x	x

Supplementary Table 4: Diagnostic predictions based on the TransCUPtomics classifiers for tumors analyzed by RNAseq. For each sample, the two first predictions are reported for both KNN and RF classifiers. Abbreviations: KNN: k nearest neighbor; RF: Random Forest.

Patient ID	1st KNN Prediction	KNN Probability score (1st prediction)	2nd KNN Prediction	KNN Probability score (2nd prediction)	1st RF Prediction	RF Probability score (1st prediction)	2nd RF Prediction	RF Probability score (2nd prediction)
**CUPKID-1**	Kidney renal papillary cell carcinoma	0,64	Kidney renal clear cell carcinoma	0,269	Kidney renal papillary cell carcinoma	0,166	Uterine corpus endometrial carcinoma	0,154
**CUPKID-4**	Dedifferentiated liposarcoma	0,203	Undifferentiated pleomorphic sarcoma	0,111	Bladder urothelial carcinoma	0,076	Uterine corpus endometrial carcinoma	0,07
**CUPKID-5**	Kidney renal papillary cell carcinoma	0,409	Kidney renal clear cell carcinoma	0,303	Kidney renal papillary cell carcinoma	0,166	Kidney renal clear cell carcinoma	0,082
**CUPKID-6**	Kidney renal clear cell carcinoma	0,424	Kidney renal papillary cell carcinoma	0,387	Kidney renal papillary cell carcinoma	0,174	Kidney renal clear cell carcinoma	0,1
**CUPKID-7**	Kidney renal papillary cell carcinoma	0,595	Kidney renal clear cell carcinoma	0,405	Kidney renal papillary cell carcinoma	0,278	Kidney renal clear cell carcinoma	0,194
**CUPKID-8**	Kidney renal clear cell carcinoma	0,908	Kidney renal papillary cell carcinoma	0,092	Kidney renal clear cell carcinoma	0,28	Kidney renal papillary cell carcinoma	0,214
**CUPKID-9**	Kidney renal papillary cell carcinoma	0,694	Kidney renal clear cell carcinoma	0,306	Kidney renal papillary cell carcinoma	0,342	Kidney renal clear cell carcinoma	0,33
**CUPKID-19**	Kidney renal papillary cell carcinoma	0,898	Kidney renal clear cell carcinoma	0,102	Kidney renal papillary cell carcinoma	0,414	Kidney renal clear cell carcinoma	0,116
**CUPKID-20**	Kidney renal clear cell carcinoma	0,311	Undifferentiated pleomorphic sarcoma	0,224	Kidney renal papillary cell carcinoma	0,154	Kidney renal clear cell carcinoma	0,12
**CUPKID-21**	Kidney renal clear cell carcinoma	0,602	Kidney renal papillary cell carcinoma	0,398	Kidney renal clear cell carcinoma	0,454	Kidney renal papillary cell carcinoma	0,284
**CUPKID-22**	Kidney renal clear cell carcinoma	0,786	Kidney renal papillary cell carcinoma	0,214	Kidney renal clear cell carcinoma	0,126	Kidney renal papillary cell carcinoma	0,098
**CUPKID-25**	Kidney renal papillary cell carcinoma	0,885	Kidney renal clear cell carcinoma	0,115	Kidney renal papillary cell carcinoma	0,348	Kidney renal clear cell carcinoma	0,222

Supplementary Table 5: Pathological characteristics of all samples and final proposed diagnoses based on the combination of pathological and molecular features.

Patient ID	Sample origin	Pathological analysis	Cytological aspect	Architectural aspect	Peritumoral stroma	Positive IHC staining or evidence of FH or INI-1 deficiency	Negative IHC staining or persistence of FH or INI-1 expression	PDL-1 expression (CPS)	Pathological conclusion	Key molecular features	Final diagnosis
**CUPKID-1**	Nephro - adrenalectomy	Centralized review	Eosinophilic cells	Cohesive cells forming nests or tubules	Fibrosis with plasma cells and lymphocytes	AE1/AE3, EMA, PAX8, WT1, CA9,TFE3	CK7, p63, SF1, TTF1,GATA3, PS100, MelanA, ERG ,CD31, CD21, RO, RP, p16, ALK, Glypican-3, Bcl10, BAP1 Calretinin, Calcitonin, FH+, INI-1+	NA	Morphological aspect compatible with a renal origin and IHC stainings suggestive of clear-cell renal cell carcinoma	TransCUPtomics: renal origin Methylation Analysis: renal origin *SETD2*, *NF2* and BA*P1* mutations *CDKN2A* homozygous deletion	KIRC
**CUPKID-2**	Bone metastasis resection	Local	Clear cells	Aspect of undifferentiated carcinoma	NA	AE1/AE3, PAX8, CA8, HNF1b	CK7, CK20, WT1, p53, PR	NA	Morphological aspect and IHC stainings suggestive of clear-cell renal cell carcinoma	Methylation Analysis: renal origin *NF2* and *VHL* mutations *CDKN2A* homozygous deletion	KIRC
**CUPKID-3**	Adrenal gland biopsy	Centralized review	Large cells with clear or eosinophilic cytoplasm	Cohesive cells forming nests or tubules, tumor necrosis	Inflammatory stroma with plasma cells, lymphocytes, macrophages and polymorphonuclear neutrophils	AE1/AE3, PAX8, CA9	CK7, CK20, GATA3 MelanA, Calretinin, Synaptophysin	NA	Morphological aspect and IHC stainings suggestive of clear-cell renal cell carcinoma	Methylation Analysis: renal origin *SETD2* and *PBRM1* mutations *CDKN2A* homozygous deletion *TFEB* focal amplification	*TFEB*-amplified RCC

**CUPKID-4**	Lymph nodes dissection	Centralized review	Eosinophilic cells	Undifferentiated carcinoma with sheets of uncohesive cells, tumor necrosis	Inflammatory stroma with lymphocytes	AE1/AE3, Vimentin, EMA, p16, E-cadherin, loss of INI-1 expression, GATA3, CK19, RP	CK7, CK20, CK5/6, p63, p40, PS100,SALL4, SOX10, RO, MelanA, Chromogranin A, Calcitonin, Thyroglobulin, WT1, CD56, TTF1, HMB45, Her2, PAX, GCDFP15	NA	Morphological aspect consistent with the diagnosis of renal medullary carcinoma, supported by the loss of INI-1 expression.	TransCUPtomics: No diagnosis proposed Methylation Analysis: RMC cluster *SMARCB1* (*INI-1*) inactivating translocation	Renal Medullary Carcinoma
**CUPKID-5**	Brain metastasis resection	Centralized review	Atypical celsl with irregular sizes and nuclear aspects	Aspect of undifferentiated carcinoma with cohesives cells forming nests	Hemorrhagic stroma with lymphocytes	AE1/AE3,CK20, PAX8, CD10 PS100, SATB2, EMA, MelanA (Low and heterogenous)	CK7, Glypican-3, RP, Oligo2, SALL4, CD30, GFAP, TTF1, CD2, p40, Thyroglobulin, NKX3.1, CA9, FH+, TFE3	NA	Morphological aspect and IHC stainings compatible with a renal origin	TransCUPtomics: renal origin Methylation Analysis: renal origin *PBRM1* and *STAG2* mutations	URCC
**CUPKID-6**	Bone biopsy	Local	Clear or eosinophilic cylindrical cells with irregular nuclei	Cohesive cells forming nests or tubules	Fibrous stroma. Low immune cell infiltration.	CK7, PAX8	CK20, TTF1, GATA3, CDX2	NA	Morphological aspect and IHC staining compatible with a renal origin	TransCUPtomics: renal origin Methylation Analysis: renal origin *NF2* mutation *CDKN2A* homozygous deletion	URCC
**CUPKID-7**	Bone biopsy	Centralized review	Large cells with clear or oesinophilic cytoplasm	Cohesive cells arranged in nests or tubules, sarcomatoid component with spindled and less coehsive cells	Fibrosis with lymphocytes	AE1/AE3, PAX8, CA9, WT1, MelanA (Low and heterogenous)	GATA3, PSAP, TTF1, calretinin, TFE3	NA	Morphological aspect and IHC stainings compatible with a renal origin with a sarcomatoid component.	TransCUPtomic: renal origin Methylation Analysis: renal origin *NF2* and *SETD2* mutations	URCC

**CUPKID-8**	Lymph node biopsy	Centralized review	Atypical cells with eosinophilic cytoplasm and eccentric nuclei realizing a rhabdoid aspect	Uninformative architectural aspect due to lack of material	Fibrous stroma. Low immune cell infiltration.	CK20, SATB2, PAX8	CK7, CDX2, TTF1, FH+, INI1+	NA	Morphological aspect and IHC stainings compatible with a renal origin	TransCUPtomics: renal origin	URCC
**CUPKID-9**	Lymph node biopsy	Centralized review	Clear cells	Large area of cohesive cells arranged in nests with a sarcomatoid component, tumor necrosis	Inflammatory stroma with prominent capillary network, lymphocytes, macrophages and polynuclear neutrophils.	AE1/AE3, CK8/18, EMA, CK19, CD10, PAX8, AMACR, GATA3, Vimentin	CK7, CK20,p40, PSA, HepPAR1, TTF1, CDX, pS100, HER2, SALL4, CD56, Synaptophysin,CD79a Chromogranin A, CD3, CD20	>50%	Morphological aspect and IHC stainings suggestive of a renal origin, probably a clear cell renal cell carcinoma	TransCUPtomics: renal origin	URCC
**CUPKID-10**	Bone biopsy	Centralized review	Eosinophilic and cylindrical cells	Cohesive cells forming nests or tubules, tumor necrosis	Fibrous and hemorrhagic stroma, with a dense network of capillaries and lymphocytes	CK7, AE1/AE3, CD10, PAX8, Vim, AMACR, Alcian blue	CK20, PSA, TTF1, ALK, PDL-1,Thyroglobulin,ERG, HMB45, CD5, MelanA, Calretinin,WT1, FH+	<5%	Morphological aspect and IHC stainings suggestive of a renal origin, probably a papillary renal cell carcinoma	NA	KIRP
**CUPKID-11**	Lymph node biopsy	Centralized review	Large cells with clear cytoplasm	Undifferentiated carcinoma with cohesive cells forming nests and a sarcomatoid component	Fibrous and inflammatory stroma with lymphocytes and plasmocytes	AE1/AE3, PAX8, CD10, Vimentin	CK7, CK20, PS100, CD45 PLAP CDX2, CK19, PSA, TTF1, Calcitonin, Thyroglobulin, RCC , p40, bHCG, TFE3, non altered *TFEB* (FISH)	<5%	Morphological aspect and IHC stainings compatible with a renal origin	NA	URCC
**CUPKID-12**	Lymph node biopsy	Centralized review	Clear cells	Cohesive cells forming nests, tumor necrosis	Prominent vascularization with a dense network of capillaries and polymorphonuclear neutrophils	AE1/AE3, CA9, PAX8, Vimentin, CD10	CK7, CK20, p63, HMB45, MelanA, Sox10, PS100, Ber-EP4, CD138, CD45, EMA, ERG, GATA3, MUM1, SATB2, TTF1, CD34, CD20, CD21, CD30, CD68, CD163	NA	Morphological aspect and IHC stainings suggestive of clear cell renal cell carcinoma	NA	KIRC

**CUPKID-13**	Lymph node biopsy	Centralized review	Large cells with clear or eosinophilic cytoplasm	Aspect of undifferentiated carcinoma with isolated cells and cells arranged in nests	NA	CK7, AE1/AE3, PAX8, BERP4, p53, BRG1, EMA	CK20, TTF1, CD10, Thyroglobulin, AFP, PLAP , SALL4, WT1, p40, ALK2, GCDFP15, Mammaglobin, GATA3, NAPSIN-1, RCC, CK5/6, p63, bHCG, OCT3/4, CD30, CKIT, Calretinin.	NA	Morphologica l aspect and IHC stainings compatible with a renal origin	NA	URCC
**CUPKID-14**	Lymph node biopsy	Local	NA	NA	Fibrous and inflammatory stroma with necrotic area. Plasmocytes, lymphocytes and macrophage infiltration.	AE1/AE3	NA	NA	Morphologica l aspect and IHC stainings suggestive of a papillary renal cell carcinoma	NA	KIRP
**CUPKID-15**	Bone biopsy	Local	Large cells with clear cytoplasm and nuclear pleomorphism	Cohesive cells forming nests and glandular structures	Fibrous stroma with necrotic areas	CA9, AMACR	CK7, CK20, PSA	NA	Morphologica l aspect and IHC stainings suggestive of a clear cell renal cell carcinoma	NA	KIRC
**CUPKID-16**	Bone biopsy	Centralized review	Large cells with clear cytoplasm	Cohesive cells forming nests	Dense vascularization	CA9, EMA, Vimentin	CK7, CK20, TTF1, ACE	NA	Morphologica l aspect and IHC stainings suggestive of a clear cell renal cell carcinoma	NA	KIRC
**CUPKID-17**	Retroperitoneal biopsy	Centralized review	Spindle-shaped cells with atypical, hyperchromati c nuclei and numerous mitotic figures Focal aspect of clear epithelial cell	Aspect of sarcomatoid carcinoma	Fibrous and inflammatory stroma	AE1/AE3, PAX8, CA9, EMA	CK7, CK20, PS100 , AMACR, MDM2	NA	Morphologica l aspect and IHC stainings suggestive of a clear cell renal cell carcinoma with a sarcomatoid component	NA	KIRC

**CUPKID-18**	Bone biopsy	Centralized review	Large cells with clear cytoplasm with irregular nuclei	Papillary aspect with cohesive cells and tubules	Fibrous and inflammatory stroma	AE1/AE3, PAX8, CA9, CD10, EMA, AMACR	CK7, CK20, TTF1, GATA3, p40, Thyroglobulin, OCT4, CD30, Synaptophysin, SOX10, ACE, Alcian blue, MelanA, HMB45, CK19, GFA, PS100, Calretinin	NA	Morphological aspect and IHC stainings compatible with a renal origin	NA	URCC
**CUPKID-19**	Lymph node dissection	Local	Large cylindrical cells with clear or eosinophilic cytoplasm	Papillary aspect with cohesive cells arranged in tubules, tumor necrosis	Fibrous stroma	AE1/AE3, PAX8, CA9, AMACR	CK7, CK20, p63, CDX2, GATA3, TTF1, PS100, HMB45, NKX3.1, P40, CD45, p16,CK5-6-, P40-, P63-, calrétinin, synaptophysin, chromogranin, actin, ERG, LMP, CD20, calcitonin, WT1, thyroglobulin, SOX10, CD99, CD117, CD30, BCL2	20%	Morphological aspect and IHC stainings compatible with a renal origin	TransCUPtomics: renal origin *NF2* and *B2M* mutations	URCC
**CUPKID-20**	Bone biopsy	Centralized review	Cylindrical eosinophilic or clear cells	Cohesive cells forming nests or tubules, tumor necrosis	Fibrous and inflammatory stroma with polymorphonuclear neutrophils	AE1/AE3, PAX8, Vimentin, AMACR	CK7, CK20, p63, Sox10, CDX2, chromogranin, synaptophysin, GATA3, TTF1,CD34, PS100, desmin, NUT, RO, SALL4, HMB45, CD5, CD10, CA9, CD117 TFE3, P63, thyroglobulin, calcitonin, NKX3.1, INI1+, BRG1+	<5%	Morphological aspect and IHC stainings compatible with a renal origin	TransCUPtomics: renal origin *NF2* mutation *CDKN2A* homozygous deletion	URCC
**CUPKID-21**	Bone biopsy	Local	Large cells with clear cytoplasm, tumor necrosis	Aspect of undifferentiated carcinoma with cohesive cells	Fibrous stroma	AE1/AE3, PAX8, CA9, AMACR	CK7, CK20, TTF1, p63	NA	Morphological aspect and IHC stainings suggestive of a renal-cell clear cell carcinoma	TransCUPtomics: renal origin	KIRC

**CUPKID-22**	Bone biopsy	Centralized review	Clear or eosinophilic cells of various sizes	Aspect of undifferentiated carcinoma with a sarcomatoid component, tumor necrosis	Inflammatory stroma with plasmocytes and lymphocytes	AE1/AE3, CK8/18, CK20, PAX8, SATB2, GATA3, CD117	CK7, CA9, AMACR, S100, SALL4, TTF1, SOX10, PSA, PSAP, NKX3.1 , ChromograninA, Synaptophysin, INSM1, CDX2, SMARCA4+	NA	Morphological aspect and IHC stainings compatible with a renal origin	TransCUPtomics: renal origin *NF2* and *SMARCB1* mutations	URCC
**CUPKID-23**	Bone biopsy	Centralized review	Large cylindrical cells with eosinophilic cytoplasm	Aspect of undifferentiated carcinoma with cohesive cells	Fibrous and inflammatory stroma	AE1/AE3, PAX8, AMACR	CK7, CK20, TTF1, CA9, HMB45, MelanA, INI-1+	NA	Morphological aspect and IHC stainings compatible with a renal origin, possibly a papillary renal cell carcinoma	NA	URCC
**CUPKID-24**	Lymph node dissection	Centralized review	Atypical cells of irregular shapes and sizes with eosinophilic cytoplasm and large irregular nuclei	Heterogenous histological aspect with glandular structures and uncohesive papillae or micro-papillae	Fibrous stroma with necrotic areas	AE1/AE3, EMA, CK7, GATA3, BerEP4	CK20, TTF1, Mammaglobin, CDX2, HER2, p63, PAX8, SOX10, PS100, CK5/6, D240, SALL4, WT1 TRPS1, RO, RP, CK19, HBME, SMARCA4+, BAP1+	12%	Morphological aspect and IHC stainings compatible with a renal origin	RNA Panel : PRCC::TFE3	TFE3-rearranged RCC
**CUPKID-25**	Lymph node biopsy	Local	Large cells with irregular shapes and sizes and clear or eosinophilic cytoplasm	Aspect of undifferentiated carcinoma with cohesive cells	Abondant necrosis and polymorphonuclear neutrophils infiltration	AE1/AE3, CK19, PAX8, CA9, CD138	CK7, CK20, CK903, EMA, p40, p63, Calretinin, WT1, CD117, TTF1, Thyroglobulin, PAP, CD30, CD20, CD5, CD45, SOX10, Synaptophysin, CD56	NA	Morphological aspect and IHC stainings suggestive of a renal origin	TransCUPtomics: renal origin HNF1A mutation CDKN2A/B and MTAP deletion	KIRP

Supplementary Table 6: Therapeutic management and survival of all patients. Abbreviations: KIRC: kidney clear cell carcinoma; KIRP: kidney papillary carcinoma; TFEB-amp RCC: TFEB-amplified renal cell carcinoma; RMC: renal medullary carcinoma; URCC: undifferentiated renal cell carcinoma; CRT: chemoradiation therapy: RT: radiation therapy; PFS: progression-free survival; OS: overall survival; PR: partial response; SD: stable disease; PD: progressive disease; ANOD: alive with no evidence of disease; AD: alive with disease; DOD: dead of disease; NA: not applicable. * Censored at time of publication.

CUPKID ID	Final diagnosis	Locoregional treatment	Distant relapse after locoregional treatment	First-line systemic treatment	PFS1 (months)	Best response under first-line systemic treatment	Second-line systemic treatment	PFS2 (months)	Best response under second-line systemic treatment	Third-line systemic treatment	PFS3 (months)	Best response under third-line systemic treatment	Fourth-line systemic treatement	PFS4 (months)	Best response under fourth-line systemic treatment	Final status	Overall Survival (months)
**CUPKID-1**	KIRC	Nephro-adrenalectomy, Cryotherapy	No	NA	NA	NA	NA	NA	NA	NA	NA	NA	NA	NA	NA	ANOD	33
**CUPKID-2**	KIRC	NA	NA	Pembrolizumab+Axitinib	16,7	SD	Cabozantinib	1,0	SD	Pembrolizumab+ Lenvatinib	3,9	PD	NA	NA	NA	AD	27,7*
**CUPKID-3**	TFEB	NA	NA	Pembrolizumab+Axitnib	21,9*	PR	NA	NA	NA	NA	NA	NA	NA	NA	NA	AD	22,7*
**CUPKID-4**	RMC	Lymph nodes resection, adjuvant CRT	No	NA	NA	NA	NA	NA	NA	NA	NA	NA	NA	NA	NA	ANOD	39
**CUPKID-5**	URCC	Brain metastasis resection, adjuvant RT	Bones	Sunitinib	15,2*	SD	NA	NA	NA	NA	NA	NA	NA	NA	NA	AD	47,9*
**CUPKID-6**	URCC	NA	NA	Axitinib	4,8	PD	NA	NA	NA	NA	NA	NA	NA	NA	NA	DOD	6,7
**CUPKID-7**	URCC	NA	NA	Carboplatin+Paclitaxel	3,3	PD	Pembrolizumab+Axitinib	2,0	PD	Cabozantinib	10,9	PR	Sunitinib	4,4*	SD	AD	24,4*
**CUPKID-8**	URCC	NA	NA	Carboplatin+Paclitaxel	5,2	SD	Nivolumab	20,2	PD	Carboplatin+ Pemetrexed	1,2	PD	NA	NA	NA	DOD	30,6
**CUPKID-9**	URCC	NA	NA	Nivolumab+Ipilimumab	1,0	PD	NA	NA	NA	NA	NA	NA	NA	NA	NA	DOD	5,1
**CUPKID-10**	KIRP	Laminectomy, adjuvant RT	Bones	Cabozantinib	6,3	SD	Nivolumab	1,4	PD	Axitinib	4,7	SD	NA	NA	NA	DOD	52,3
**CUPKID-11**	URCC	NA	NA	Nivolumab+Ipilimumab	1,7	PD	Cabozantinib	1,8	PD	Gemcitabine	1,7	PD	NA	NA	NA	DOD	7,6
**CUPKID-12**	KIRC	NA	NA	Pembrolizumab+Axitnib	7,5	SD	Cabozantinib	2,6	PD	NA	NA	NA	NA	NA	NA	DOD	18,5
**CUPKID-13**	URCC	NA	NA	Sunitinib	5,2	SD	Cabozantinib	2,2	SD	Axinitib	0,3	PD	NA	NA	NA	DOD	9,3
**CUPKID-14**	KIRP	NA	NA	Nivolumab+Ipilimumab	1,3	PD	Axitinib	19,2	PR	Cabozantinib	10,7	SD	NA	NA	NA	AD	50,9*
**CUPKID-15**	KIRC	NA	NA	Temsirolimus	9,0	SD	Sunitinib	7,0	SD	NA	NA	NA	NA	NA	NA	DOD	19,7
**CUPKID-16**	KIRC	NA	NA	Sunitinib	130,1*	PR	NA	NA	NA	NA	NA	NA	NA	NA	NA	AD	134,2*
**CUPKID-17**	KIRC	NA	NA	Nivolumab+Ipilimumab	2,1	PD	Cabozantinib	12,7	PR	NA	NA	NA	NA	NA	NA	DOD	18,3
**CUPKID-18**	URCC	NA	NA	Nivolumab+Ipilimumab	1,3	PD	Cabozantinib	4,4	PD	NA	NA	NA	NA	NA	NA	DOD	9,5
**CUPKID-19**	URCC	NA	NA	Cisplatin+Gemcitabine	10,7	PR	Pembrolizumab	0,9	PD	Carboplatin+Paclitaxel	1,1	PD	Cabozantinib	1	SD	AD	19,4*

**CUPKID-20**	URCC	NA	NA	Cabozantinib+Nivolumab	1,8	PD	Carboplatin+Paclitaxel	3*	PR	NA	NA	NA	NA	NA	NA	AD	6,2*
**CUPKID-21**	KIRC	NA	NA	Cisplatin+Gemcitabine	7,9	PR	Carboplatin+Paclitaxel	1,0	PD	Everolimus	2,1	PD	NA	NA	NA	DOD	15,3
**CUPKID-22**	URCC	NA	NA	Nivolumab+Ipilimumab	1,4*	SD	NA	NA	NA	NA	NA	NA	NA	NA	NA	AD	3,3*
**CUPKID-23**	URCC	NA	NA	Cabozantinib+Nivolumab	10,8*	PR	NA	NA	NA	NA	NA	NA	NA	NA	NA	AD	11*
**CUPKID-24**	TFE3	Lymph nodes resection, adjuvant RT	Lymph nodes	Carboplatin+Paclitaxel	7,3	PR	Pembrolizumab	7,4*	SD	NA	NA	NA	NA	NA	NA	AD	36,4*
**CUPKID-25**	KIRP	NA	NA	Cisplatin+Gemcitabine	4,9	PR	Paclitaxel	2,1	PD	Cabozantinib	7,1	PR	NA	NA	NA	AD	15,5*
